# Gut microbiota-derived indole-3-acetic acid suppresses high myopia progression by promoting type I collagen synthesis

**DOI:** 10.1038/s41421-024-00709-5

**Published:** 2024-08-27

**Authors:** Hao Li, Yu Du, Kaiwen Cheng, Yuxi Chen, Ling Wei, Yujun Pei, Xiaoyu Wang, Lan Wang, Ye Zhang, Xiaoxin Hu, Yi Lu, Xiangjia Zhu

**Affiliations:** 1 Eye Institute and Department of Ophthalmology, Eye & ENT Hospital, Fudan University, Shanghai, China; 2 State Key Laboratory of Medical Neurobiology, Fudan University, Shanghai, China; 3 Department of Ophthalmology, Zhongshan Hospital, Fudan University, Shanghai, China; 4 Key Laboratory of Myopia and Related Eye Diseases, NHC; Key Laboratory of Myopia and Related Eye Diseases, Chinese Academy of Medical Sciences, Shanghai, China; 5 Shanghai Key Laboratory of Visual Impairment and Restoration, Shanghai, China; 6 Mass Spectrometry Platform, Institutes of Biomedical Sciences, Fudan University, Shanghai, China

**Keywords:** Cell signalling, Transcription

## Abstract

High myopia (HM) is a leading cause of blindness worldwide with currently no effective interventions available. A major hurdle lies in its often isolated perception as a purely ocular morbidity, disregarding potential systemic implications. Recent evidence suggests the existence of a gut-eye axis; however, the role of gut microbiota in the pathogenesis of HM remains largely unexplored. Herein, we provide a potential crosstalk among HM’s gut dysbiosis, microbial metabolites, and scleral remodeling. Utilizing 16S rRNA gene sequencing, we observed an altered gut microbiota profile in HM patients with a significant reduction in probiotic abundance compared with healthy controls. Subsequent targeted metabolic profiling revealed a notable decrease in plasma levels of the gut microbiota-derived metabolite indole-3-acetic acid (3-IAA) among HM patients, which is closely associated with the reduced probiotics, both negatively correlated with HM severity. Genetic analyses determined that gut microbiota are causally associated with myopia risk. Importantly, when mice subjected to HM modeling receive fecal microbiota transplantation from healthy donors, there is an increase in 3-IAA plasma levels and simultaneous retardation of HM progression along with better maintenance of collagen type I alpha 1 (COL1A1) expression in the sclera. Furthermore, 3-IAA gavage achieves similar effects. Mechanistic investigations confirm the transcriptional activation of COL1A1 by 3-IAA via promoting the enrichment of SP1 to its promoter. Together, our findings provide novel insights into the gut microbiota-eye axis in the pathogenesis of HM and propose new strategies for HM intervention by remodeling the gut microbiota and indole supplementation.

## Introduction

High myopia (HM), defined as a spherical equivalent ≤ –6.00 diopters (D) or an axial length (AL) ≥ 26 mm, represents a more severe form of myopia and poses a higher risk for glaucoma, cataracts, and macular degeneration^1,2^. Unfortunately, no proven effective or safe treatments are currently available for HM patients. Thus, it is imperative to elucidate the underlying mechanisms and explore effective strategies for delaying the onset and progression of HM.

The characteristic feature of HM is the excessive elongation of the eye’s AL, primarily attributed to scleral thinning. Previous studies have indicated that extracellular matrix (ECM) remodeling, specifically involving type I collagen, the main ECM component, contributes to the decline in scleral thickness, which weakens the structural framework of the sclera and induces myopia^3–5^. Epidemiological studies have supported multiple environmental risk factors for HM. Over the past two decades, the gut microbiota has been established as a pivotal environmental factor in maintaining overall body homeostasis^6,7^. Notably, recent reports have linked microbiota to collagen remodeling and fibrosis in various diseases. For instance, restoration of healthy gut microbiota in germ-free mice has been shown to improve cardiac function, prevent excessive fibrosis, and restore ECM integrity under stress conditions^8^. Additionally, *Bacteroides fragilis* has been found to ameliorate renal fibrosis in mice by inhibiting oxidative stress and inflammation^9^. Moreover, the connection between gut microbiota and ocular diseases has garnered increasing attention, with evidence implicating the gut microbiome in the onset and progression of conditions such as uveitis and age-related macular degeneration^10,11^. Nevertheless, the potential role of the gut-eye axis in HM remains unexplored.

The interactions between gut microbiota and the host primarily occur via gut microbiota-derived metabolites. These microbial metabolites are small molecules generated during microbial metabolism, including short-chain fatty acids, secondary bile acids, and tryptophan metabolites^12,13^. Microbial metabolites play a critical role in maintaining tissue homeostasis. Microbiota-derived acetate and propionate have been identified to attenuate fibrosis and reverse ECM disarray after transverse aortic constriction, highlighting the regulatory role of microbial metabolites in ECM remodeling. In recent years, evidence has emerged suggesting a potential link between HM and systemic metabolism. Individuals with HM showed distinct serum metabolomic profiles from those with low myopia^14,15^. Besides, supplementation with ω-3 polyunsaturated fatty acids has demonstrated protective effects against axial elongation^16^. These findings underscore the potential significance of metabolites in HM. We therefore hypothesized that the gut microbiome might participate in the development of HM via certain metabolites by influencing collagen remodeling.

In this study, we applied 16S rRNA gene sequencing and metabolomic analyses to investigate the characteristics of the gut microbiota and related metabolites of HM individuals and their links to HM phenotype. In doing so, we highlight a gut-derived tryptophan metabolite, indole-3-acetic acid (3-IAA) in association with HM severity, with fecal microbiota transplantation (FMT) in mice corroborating its inhibitory effect on HM progression. Further mechanistic investigations revealed that 3-IAA promoted the expression of collagen type I alpha 1 (COL1A1) in the sclera by enhancing the enrichment of the transcription factor SP1 in the promoter region of COL1A1. The elucidation of the mechanisms underlying the scleral collagen regulation by gut microbiota-derived metabolite may hold promise for developing novel preventive approaches and therapeutics for HM patients.

## Results

### Baseline characteristics of the study population

The clinical characteristics of the healthy controls (HC) and high myopes are summarized in Table 1. Additionally, the clinical characteristics of myopes with 24.50 ≤  AL < 26.00 mm are detailed in Supplementary Table S1. Both the discovery and validation cohorts were well-matched in terms of age, sex, body mass index (BMI), and educational level. Furthermore, no significant differences were observed in fasting blood glucose or biochemical measurements including liver and kidney function. Specifically, significant differences were observed in the mean AL, refractive error, anterior chamber depth (ACD), and lens thickness (LT) between the HM and HC subjects.

**Table 1:** Clinical characteristics of the study population.

Characteristics	Discovery set (*n* = 97)			Validation set (*n* = 49)		
	HC group (*n* = 45)	HM group (*n* = 52)	*P*-value	HC group (*n* = 23)	HM group (n = 26)	*P-*value
Age (years)	33.00 (25.50, 47.00)	31.00 (24.00, 43.25)	0.26	24.57 ± 3.62	24.41 ± 1.62	0.84
Males/Females *n* (%)	19 (42.22)/26 (57.78)	31 (59.62)/21 (40.38)	0.09	9 (39.13)/14 (60.87)	16 (61.54)/10 (38.46)	0.12
Educational level (a/b/c/d/e/f/g)	4/8/13/2/5/11/2	4/7/11/3/7/17/3	0.94	0/0/0/1/10/10/2	0/0/0/0/7/15/4	0.38
BMI (kg/m^2^)	22.03 ± 2.21	22.06 ± 2.31	0.95	20.94 (19.13, 23.38)	21.70 (20.20, 22.89)	0.40
AL (mm)-OD	23.58 (23.14, 24.12)	27.01 (26.58, 27.74)	< 0.001	23.79 (23.50, 24.12)	26.78 (26.33, 27.37)	< 0.001
AL (mm)-OS	23.42 (23.06, 24.06)	27.18 (26.51, 28.10)	< 0.001	23.78 ± 0.61	26.61 ± 0.70	< 0.001
ACD (mm)-OD	3.25 ± 0.37	3.67 ± 0.26	< 0.001	3.23 ± 0.29	3.77 ± 0.21	< 0.001
ACD (mm)-OS	3.25 ± 0.37	3.67 ± 0.26	< 0.001	3.51 ± 0.30	3.77 ± 0.25	< 0.001
LT (mm)-OD	4.00 (3.73, 4.28)	3.73 (3.58, 4.02)	0.019	3.72 ± 0.19	3.55 ± 0.22	0.004
LT (mm)-OS	3.99 (3.71, 4.27)	3.71 (3.55, 4.00)	0.023	3.70 ± 0.22	3.55 ± 0.22	0.019
WTW (mm)-OD	11.95 ± 0.43	11.92 ± 0.39	0.70	12.07 ± 0.53	12.06 ± 0.33	0.99
WTW (mm)-OS	11.98 ± 0.40	11.96 ± 0.44	0.81	12.10 ± 0.52	11.99 ± 0.45	0.41
Diopter-OD	−1.00 (−1.5, 0)	−8.00 (−10.00, −6.50)	<0.001	−0.83 ± 0.68	−7.18 ± 1.45	< 0.001
Diopter-OS	−0.75 (−1.38, 0)	−8.25 (−10.00, −6.50)	<0.001	−0.89 ± 0.79	−6.87 ± 1.69	< 0.001
IOP (mmHg)-OD	14.27 ± 2.64	15.14 ± 1.85	0.06	15.28 ± 2.90	16.19 ± 2.07	0.20
IOP (mmHg)-OS	14.48 ± 2.64	15.01 ± 1.88	0.28	15.69 ± 3.11	15.49 ± 2.47	0.80
FPG (mmol/L)	4.70 (3.80, 5.00)	4.70 (4.10, 5.20)	0.18	4.42 ± 0.50	4.59 ± 0.39	0.17
TG (mmol/L)	0.82 (0.63, 1.13)	0.84 (0.62, 1.07)	0.88	0.99 (0.62, 1.01)	0.92 (0.66, 1.01)	0.55
TC (mmol/L)	4.61 (4.09, 4.96)	4.65 (4.09, 5.18)	0.58	4.63 ± 0.80	4.32 ± 0.78	0.17
AST(U/L)	19.00 (15.50, 24.50)	19.00 (15.00, 22.75)	0.64	17.61 ± 4.56	17.33 ± 4.49	0.83
ALT(U/L)	15.00 (11.00, 29.50)	16.00 (12.00, 22.75)	0.71	19.39 (10.00, 74.00)	14.93 (9.00, 17.00)	0.40
HbA1C (%)	5.30 (5.05, 5.70)	5.30 (5.03, 5.50)	0.38	5.41 ± 0.34	5.37 ± 0.24	0.61
Cr (μmol/L)	68.24 ± 16.41	73.50 ± 13.07	0.08	71.26 ± 12.93	74.22 ± 13.11	0.43
UA (μmol/L)	312.00 (263.00, 376.00)	318.50 (276.50, 377.00)	0.48	315.9 ± 90.05	371.2 ± 101.4	0.06

Data are presented as mean ± standard deviation (SD), median with interquartile range (IQR), or *n* (%). *P*-values are based on the two-sided *t*-test for variables expressed as mean ± SD, the Wilcoxon rank-sum test for variables expressed as median (IQR), and the chi-square test for variables expressed as percentages. Educational level (a/b/c/d/e/f/g): primary education/lower secondary education/upper secondary or intermediate vocational education/higher vocational education/ undergraduate education/postgraduate education/doctoral education.

*ACD* anterior chamber depth, *LT* lens thickness, *WTW* white to white, *IOP* intraocular pressure, *FPG* fasting plasma glucose, *TG* triglyceride, *TC* total cholesterol, *AST* aspartate transaminase, *ALT* alanine transaminase, *Cr* creatinine, *UA* uric acid.

### The severity of HM is associated with gut microbiota dysbiosis

**Fig. 1:**
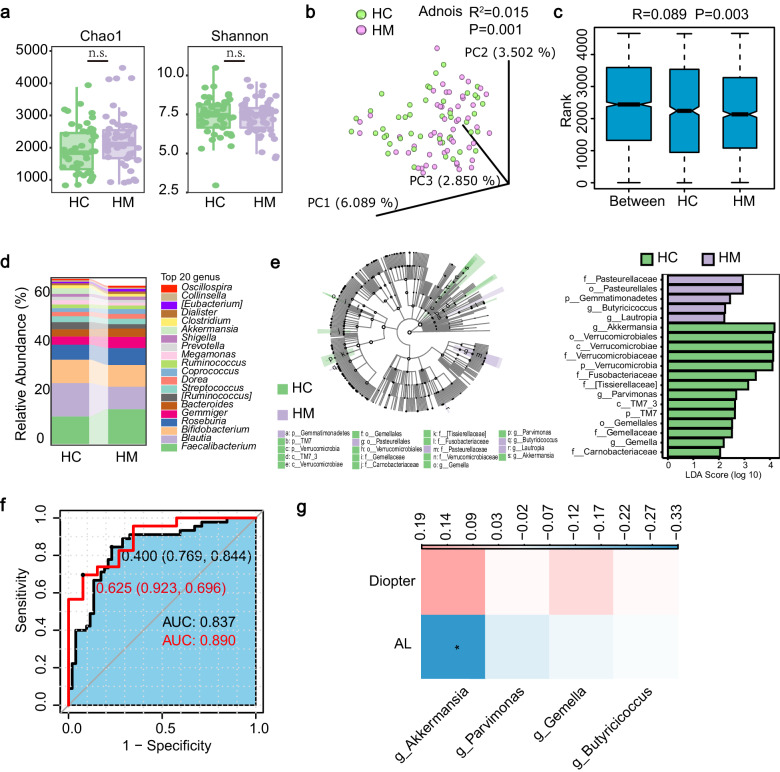
The severity of HM is associated with gut microbiota dysbiosis. **a** Alpha-diversity assessed by Chao1 (*P* = 0.23) and Shannon index (*P* = 0.84). **b** Beta-diversity assessed by PCoA based on weighted UniFrac distance. **c** Anosim analysis based on Jaccard distance. **d** Genus levels of microbial composition (top 20). **e** Upper, Cladogram of different taxonomic compositions in the HC group and HM group. Bottom, LDA effect size (LEfSe) demonstrating taxa with significantly different abundance (LDA score > 2). **f** Receiver operating characteristic curves and their corresponding AUC using a combination of discriminatory OTUs in the discovery and validation sets. **g** Heatmap showing the Spearman’s correlation coefficients among discriminatory OTUs and 2 clinical indices in an independent cohort (FDR < 0.05). For the independent cohort, *n* = 73. For the discovery set, *n* = 45 (HC), *n* = 52 (HM). For the validation set, *n* = 23 (HC), *n* = 26 (HM). The data are presented as median, quartiles, and min/max values (**a**, **c**) and evaluated using the Wilcoxon rank-sum test (**a**). *P < 0.05, **P < 0.01. n.s. not significant.

16S rRNA gene sequencing was performed on fecal samples obtained from all participants. The sequencing depth was initially validated using the dilution curve method, ensuring adequate coverage for further analysis (Supplementary Fig. S1a). Alpha-diversity indices, including Chao1 and Shannon, revealed no significant differences between HM and HC, indicating similar richness and diversity of gut microbiota in both groups (Fig. 1a). However, principal coordinate analysis (PCoA) based on the weighted UniFrac distance demonstrated a significant difference in beta diversity (Adonis: R^2^ = 0.015, *P* = 0.001), suggesting a distinct microbial composition in HM at the operational taxonomic unit (OTU) level (Fig. 1b). Analysis of Similarity (ANOSIM) further confirmed the significant shift in microbial composition (R^2^ = 0.089, *P* = 0.003), which also indicated a less heterogeneous community structure among HM individuals (Fig. 1c). Additionally, alluvial maps depicting the family (Supplementary Fig. S1b) and genus (Fig. 1d) levels of the top 20 enriched gut bacterial were generated.

To identify HM-related bacteria, biomarker species were defined using linear discriminant analysis effect size (LefSe) analysis. The results revealed that in HM group, *Akkermansia*, *Fusobacteriaceae*, *Tissierellaceae*, *Parvimonas*, *TM7-3*, *Gemella*, and *Carnobacteriaceae* were depleted, whereas *Pasteurellaceae*, *Gemmatimonadetes*, *Butyricicoccus*, and *Lautropia* were enriched (Fig. 1e). Particularly, *Akkermansia*, a probiotic with implications in various diseases, showed the highest linear discriminant analysis (LDA) score (> 4) and exhibited a significant decrease in both univariate and multivariate analyses. The discriminative power of the gut bacteria in distinguishing HM was confirmed with area under the curve (AUC) values of 0.837 in the discovery set and 0.890 in the validation set (Fig. 1f).

Further exploration into the causal relationship between myopia and gut microbiota was conducted using two-sample Mendelian randomization (MR) analysis based on large-scale public GWAS data from the FinnGen study and the GWAS Catalog, respectively^17^. Fourteen bacteria were identified with significant causal associations with myopia by two-sample MR using the inverse-variance weighted (IVW) method (Supplementary Table S2). Especially, g_*Akkermansia* emerged as a protective factor against myopia (OR = 0.797, 95% CI = 0.637–0.997, *P* = 0.047). Additionally, the MR-Egger intercept test showed no evidence of horizontal pleiotropy that might distort these results (*P* > 0.05).

In an independent cohort, we further analyzed the correlations between the abundance of altered genera and two parameters assessing the severity of myopia (AL and refractive error) and found that *Akkermansia* was negatively associated with AL (Fig. 1g).

### Healthy gut microbiota is required for the maintenance of COL1A1 expression in the sclera to retard HM

**Fig. 2:**
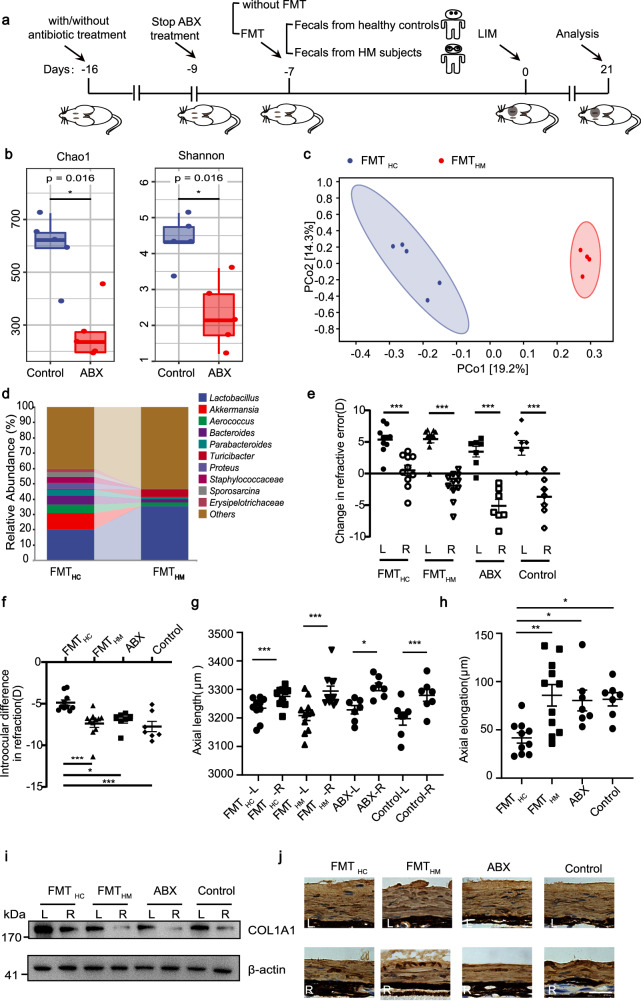
FMT delays the development of high myopia. **a** Schematic diagram of the mouse FMT process. **b** The diversity and abundance of the gut microbiota in ABX-treated and control mice were assessed by Chao1 (*P* = 0.016) and Shannon index (*P* = 0.016). **c** PCoA plot based on the Jaccard distance. **d** Profile of the gut microbiota between ABX-treated and control mice at the genus level. **e** Refraction changes of the defocused eyes (right eyes) and fellow eyes (left eyes). **f** Interocular differences in refraction in FMT_HC_ mice, FMT_HM_ mice, ABX-treated mice and control mice. **g** AL changes of defocused eyes (right eyes) or fellow eyes (left eyes) in FMT_HC_ mice, FMT_HM_ mice, ABX-treated mice, and control mice. **h** Interocular differences of AL in FMT_HC_ mice, FMT_HM_ mice, ABX-treated mice, and control mice. **i** Western blotting results depicting the COL1A1 protein expression levels in the sclera. β-actin was used as the control. **j** The protein levels of COL1A1 in sclera determined by immunofluorescence. Scale bar, 25 μm. *n* = 5 (**b**–**d**). *n* = 10 (FMT_HC_ group in **e**–**h**) and *n* = 11 (FMT_HM_ group in **e**–**h**). *n* = 7 (ABX group and control group in **e**–**h**). *n* = 4 (**i**, each sample pooled 3 sclerae). The data are presented as the mean ± SEM and evaluated using two-sided Wilcoxon rank-sum test (**e**), one-way ANOVA followed with Bonferroni’s post hoc tests (**f**, **h**) and paired *t*-test (**e**, **g**). *P < 0.05, **P < 0.01, ***P < 0.001.

To establish the causal link between gut microbiota alteration and myopia progression, we conducted FMT, transplanting feces from human to recipient C57BL/6J wild-type mice pretreated with one-week antibiotic mix (ABX) treatment (seven days) before lens-induced myopia (LIM) modeling (Fig. 2a). The efficacy of gut microbiota depletion by ABX was evaluated by 16S rRNA gene sequencing one week after ABX treatment, revealing a successful reduction in gut microbiota richness and diversity (Fig. 2b; Supplementary Fig. S2a, b). Additionally, the efficacy of transferring different microbiota compositions to mice via FMT was also examined by 16S rRNA gene sequencing: comparisons were made between FMT_HC_ mice (those administered with fecal suspension from HC human participants) and FMT_HM_ mice (those administered with fecal suspension from HM human participants). Alpha diversity analysis did not show significant differences between the FMT_HM_ and FMT_HC_ groups (Supplementary Fig. S2c, d). PCoA demonstrated a distinct separation between the FMT_HM_ and FMT_HC_ mice (Adonis: R^2^ = 0.191, *P* = 0.008, Fig. 2c). Moreover, the relative abundance of *Akkermansia*, the most significantly depleted genus in the HM group, decreased in the FMT_HM_ group compared to the FMT_HC_ group (Fig. 2d), indicating successful bacterial colonization in recipient mice.

Building on the initial assessment of treatment efficacies, LIM progression among different mouse groups was compared to investigate the influence of gut microbiota on myopia. Here, FMT_HC_ mice, FMT_HM_ mice, ABX mice (those receiving only ABX treatment without FMT), and control mice (those receiving no ABX treatment or FMT) were all subjected to LIM induction. As Fig. 2e–h displayed, mice of all groups showed significant myopic shifts and AL elongation in defocused eyes with comparable results among FMT_HM_, ABX, and control mice. However, notably, the progression of myopia was significantly attenuated in the FMT_HC_ group. Specifically, in contrast to those in the FMT_HM_ group, the refractive error was 34.0% (FMT_HM_: −7.38 D vs FMT_HC_: −4.87 D) less and the AL elongation was 51.5% (FMT_HM_: 85.79 μm vs FMT_HC_: 41.63 μm) less in the FMT_HC_ group (Fig. 2f, h). These results indicate that restoring healthy gut microbiota could alleviate the myopic progression induced by wearing a minus lens. The expression of COL1A1, the main component of the scleral ECM, exhibited decreased expression during the development of myopia (Fig. 2i, j). Remarkably, FMT_HC_ treatment significantly inhibited the reduction of scleral COL1A1 induced by HM (Fig. 2i, j; Supplementary Fig. S2e). Collectively, these results suggest that the gut microbiota plays an essential role in the development of HM.

### Gut microbiota dysbiosis results in decreased plasma level of 3-IAA

**Fig. 3:**
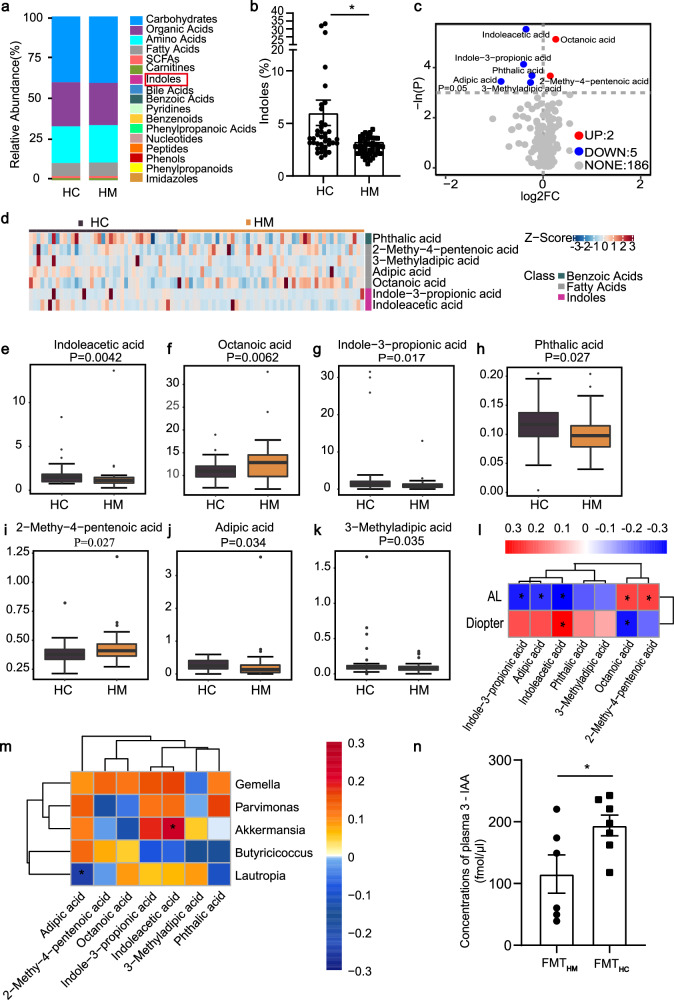
Metabolomic profiles of plasma in HM subjects. **a** Relative abundance of metabolite types among HC and HM subjects. **b** Relative abundance of indoles among HC and HM subjects (*P* = 0.0201). **c** Volcano plots showing the results of differential metabolites based on single-dimensional statistical analysis. **d** Heatmap showing the classification of plasma differential metabolites between HC and HM subjects. **e**–**k** Plot box showing the significantly changed metabolites between the HC and HM groups. **l** Heat map showing the Spearman’s correlation intensity of altered plasma metabolites with AL and diopter. **m** Heat map showing Spearman’s correlation intensity between altered plasma metabolites and significantly changed bacteria. **n** The serum concentrations of 3-IAA in FMT mice. *n* = 39 (HC group in **a**–**m**) and *n* = 49 (HM group in **a**–**m**). *n* = 6 (FMT_HC_ in **n**) and *n* = 7 (FMT_HM_ in **n**). Data are represented as the median, quartiles, and min/max values (**e**–**k**) or mean ± SEM (**c**, **e**). *P*-values were evaluated by unpaired *t*-test. *P < 0.05, **P < 0.01.

Considering the existence of the host-microbe metabolic axis^18^, we conducted targeted quantitative metabolomic profiling to further explore the plasma metabolome changes possibly related to gut microbiota dysbiosis in the discovery set. A total of 193 metabolites were identified which were subsequently categorized into 17 functional groups. Orthogonal partial least-squares discriminant analysis (OPLS-DA) did not reveal distinct clustering patterns between the HM and HC groups (Supplementary Fig. S3a, b). Among these metabolite categories, a significant decrease in the levels of indoles within the HM group was detected by relative abundance analysis (Fig. 3a, b). Further, univariate statistical analysis identified two upregulated metabolites and five downregulated metabolites in HM subjects (Fig. 3c, d). Notably, 3-IAA showed the most pronounced decrease (Fig. 3e–k).

Correlation analyses between the metabolites and myopia severity revealed negative correlations between plasma indole-3-propionic acid, adipic acid, and 3-IAA levels with AL among both HM and HC subjects, while plasma 3-IAA also exhibited a positive correlation with refractive errors (Fig. 3l). Further, a positive correlation was observed between the *Akkermansia* abundance and 3-IAA levels across both HM and HC subjects (Fig. 3m). This suggests that *Akkermansia* may play a crucial role in modulating 3-IAA levels, which align with previous studies linking this genus to indole-derived metabolites, particularly 3-IAA^19,20^.

To validate the functional relevance of these findings, we measured the plasma 3-IAA concentration in LIM mice that had received FMT from either HM or HC subjects. Interestingly, mice in FMT_HC_ group showed higher plasma 3-IAA concentrations compared to those in the FMT_HM_ group (Fig. 3n; Supplementary Fig. S3c, d). These findings confirm that the intact gut microbiota from HC subjects has a greater capacity to produce 3-IAA underscoring the potential role of 3-IAA in mediating the gut-eye axis in HM progression.

### Daily supplementation of 3-IAA alleviates the progression of HM via boosting COL1A1 expression in the sclera

**Fig. 4:**
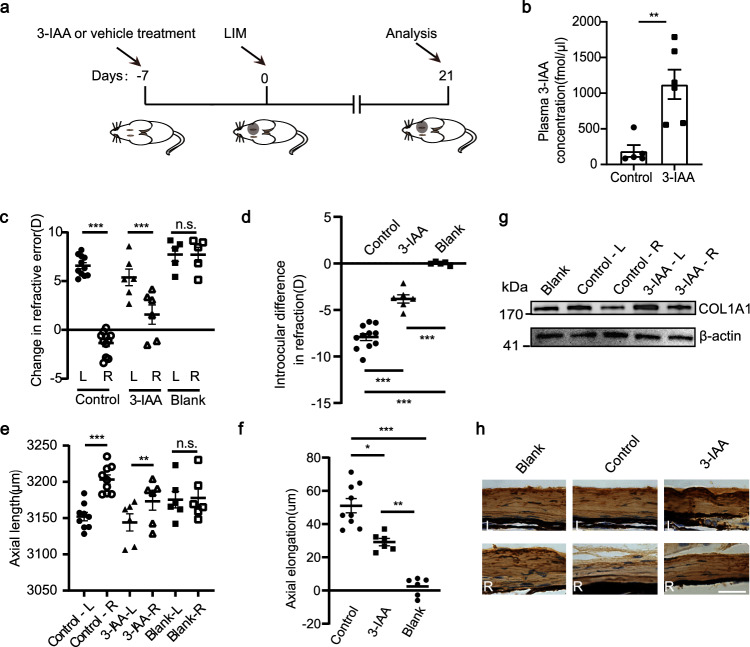
3-IAA gavage suppresses myopia progression. **a** Schematic diagram of the mouse experimental process. **b** The concentration of 3-IAA in the peripheral blood at 3 h after 3-IAA gavage. **c** Refraction changes of defocused eyes (right eyes) and fellow eyes (left eyes). **d** Interocular differences in refraction. **e** Refraction changes of the defocused eyes (right eyes) and fellow eyes (left eyes). **f** Interocular differences in AL. **g** Western blotting results of the COL1A1 protein expression levels in the sclera. β-actin was used as the control. **h** The protein levels of COL1A1 in sclera were determined by immunofluorescence. Scale bar, 25 μm. *n* = 5 (control group in **b**) and *n* = 6 (3-IAA group in **b**). *n* = 9 (FMT_HC_ group in **c**–**f**) and *n* = 6 (FMT_HM_ group and blank group in **c**–**f**). *n* = 4 (**g**, each sample pooled 3 sclerae). The data are presented as the mean ± SEM and evaluated using an unpaired *t*-test (**b**), paired *t*-test (**c**, **e**), and one-way ANOVA followed by Bonferroni’s post hoc tests (**d**, **f**). *P < 0.05, ** *P* < 0.01, ***P < 0.001. n.s. not significant.

To further investigate the effect of 3-IAA in myopia development, mice were given 3-IAA by intragastric gavage daily starting from 7 days before myopia induction, and the supplementation continued for four weeks (Fig. 4a). The 3-IAA treatment had no significant effects on refraction or body weight during normal refractive development in mice (Supplementary Fig. S4a, b). However, 3-IAA supplementation significantly increased the plasma concentration of 3-IAA from 189.1 fmol/μL to 1123 fmol/μL (Fig. 4b; Supplementary Fig. S4c), and the scleral concentration of 3-IAA from 0.0073 ng/g to 0.0145 ng/g (Supplementary Fig. S4d). Mice subjected to LIM (control group) and LIM plus 3-IAA supplement (3-IAA group) both exhibited significant myopic shifts and AL elongation in defocused eyes compared with blank groups (Fig. 4c, e). Yet, the induced myopic refractive error was 51.9% (Control: −7.92 D vs 3-IAA: −3.81 D) less and the AL elongation was 35.9% (Control: 53.30 μm vs 3-IAA: 34.17 μm) lower in the 3-IAA group compared with the control group (Fig. 4c–f). Furthermore, 3-IAA treatment attenuated the myopia-induced decrease in COL1A1 levels in the sclera of these mice (Fig. 4g, h; Supplementary Fig. S4e). These findings suggest that 3-IAA has the potential to alleviate the progression of HM.

### 3-IAA promotes the expression of COL1A1 in vitro independent of the aryl hydrocarbon receptor (AHR) pathway

**Fig. 5:**
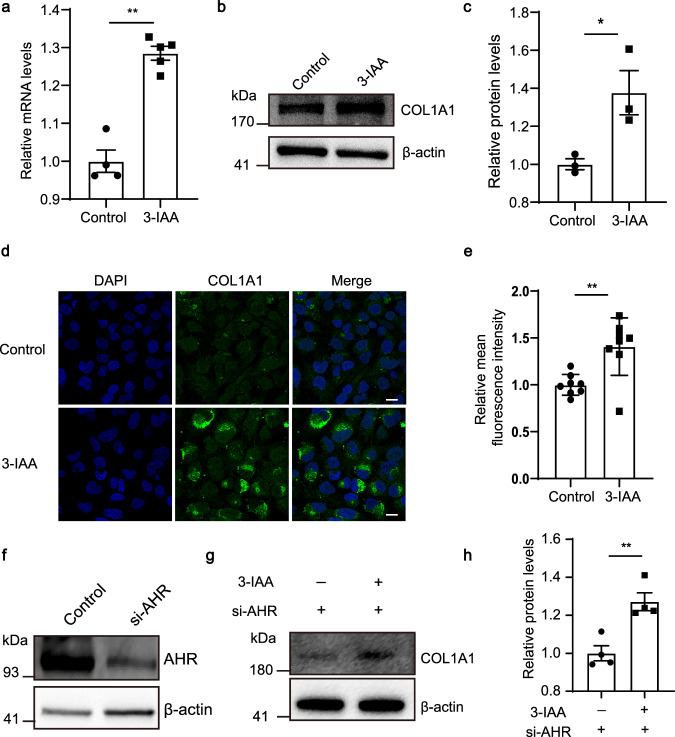
3-IAA promotes COL1A1 expression independent of AHR pathway. **a** The *COL1A1* mRNA expression levels in HFSF cells. **b**, **c** The protein levels of COL1A1 in HFSF cells were determined by western blot analysis. β-actin was used as the control. **d**, **e** Immunofluorescence analysis of COL1A1 protein expression in HFSF cells. scale bars, 20 µm. **f** The protein levels of AHR determined by western blot analysis. β-actin was used as the control. **g**, **h** The protein levels of COL1A1 in HFSF cells transfected with AHR siRNA with and without 3-IAA treatments. β-actin was used as the control. *n* = 4 (control group in **a**) and *n* = 5 (3-IAA group in **a**); *n* = 3 (**c**); *n* = 4 (**h**); The data are presented as the mean ± SEM and evaluated using an unpaired *t*-test (**a**, **c**, **e**, **h**). *P < 0.05, **P < 0.01.

To explore the effect of 3-IAA on COL1A1 expression, human fetal scleral fibroblasts (HFSFs) were treated with 3-IAA (250 μM). Significant elevation of COL1A1 expression at both mRNA (Fig. 5a) and protein levels (Fig. 5b–e) were seen upon 3-IAA treatment.

Given that 3-IAA has been reported as an endogenous ligand for the AHR^21^, we investigated whether 3-IAA promotes COL1A1 expression through the AHR pathway. We found that the mRNA expression level of *CYP1A1*, a well-known target gene of AHR, was significantly increased by 3-IAA, indicating the activation of the AHR signaling pathway in HFSFs (Supplementary Fig. S5). We then knocked down AHR using small interfering RNA (siRNA) in HFSFs (Fig. 5f), however the stimulatory effect of 3-IAA on COL1A1 expression was not abolished (Fig. 5g, h). These findings suggest that 3-IAA promotes COL1A1 expression independent of AHR signaling.

### 3-IAA transcriptionally activates COL1A1 expression by increasing the enrichment of SP1 in its promoter

**Fig. 6:**
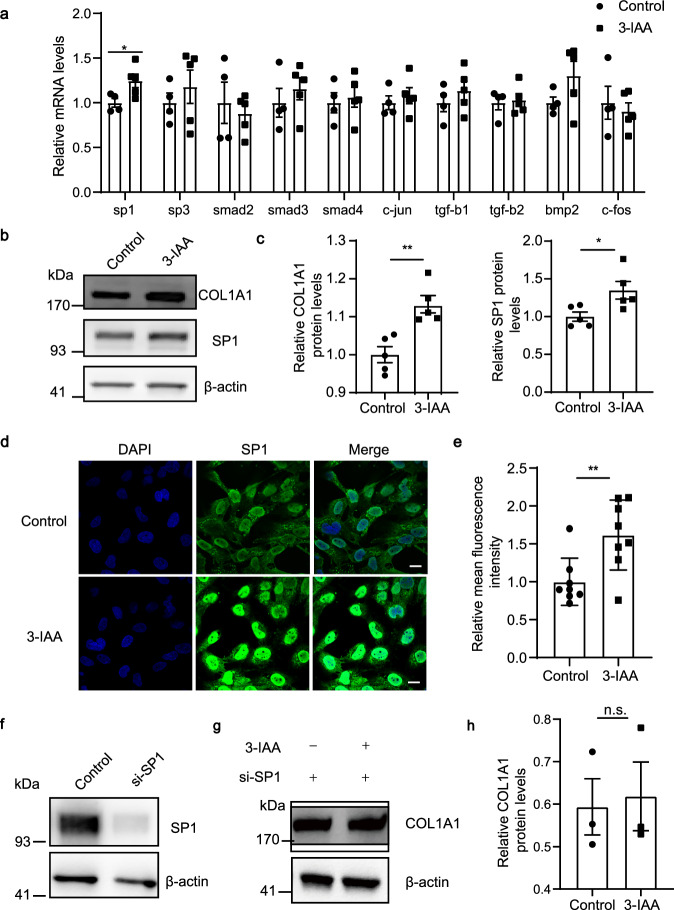
SP1 is involved in 3-IAA mediated regulation of COL1A1 expression. **a** mRNA levels of key genes in COL1A1 biosynthesis. **b**, **c** The protein levels of COL1A1 and SP1 in HFSF cells determined by western blot analysis. β-actin was used as the control. **d**, **e** Immunofluorescence analysis of SP1 protein expression in HFSF cells. scale bars, 20 µm. **f** HFSF cells were transfected with scrambled or SP1 siRNA. The protein levels of SP1 were determined by western blot. β-actin was used as the control. **g**, **h** HFSF transfected with SP1 siRNA were treated with or without 3-IAA(250 μM). The protein levels of COL1A1 in HFSF cells were determined by western blot. β-actin was used as the control. Three times each experiment was repeated independently with similar results. *n* = 4 (control group in **a**) and *n* = 5 (3-IAA group in **a**). *n* = 5 (**c**); *n* = 3 (**h**). The data are presented as the mean ± SEM and evaluated using unpaired *t*-test (**a**, **c**, **e**, **h**). *P < 0.05, **P < 0.01.

To investigate how 3-IAA regulates COL1A1 expression, we detected the expression of the key regulators involved in the regulation of COL1A1. We found that the 3-IAA remarkably increased the expression of SP1, a key transcription factor involved in collagen regulation and myopia development, at both mRNA (Fig. 6a) and protein levels (Fig. 6b–e), whereas 3-IAA treatment had no obvious effect on the mRNA expression of other regulators: *SP3*, *SMADs*, *c-JUN*, etc., in HFSFs (Fig. 6a). Inhibition of SP1 by siRNA abolished the stimulation effect of 3-IAA on the expression of *COL1A1* in HFSFs (Fig. 6h). Moreover, the scleral protein expression level of SP1 in FMT_HC_ group and 3-IAA-treated group were significantly increased compared with FMT_HM_ group and vehicle-treated group respectively, which were accompanied with a similar tendency of COL1A1 expression change (Supplementary Fig. S6a, b).

**Fig. 7:**
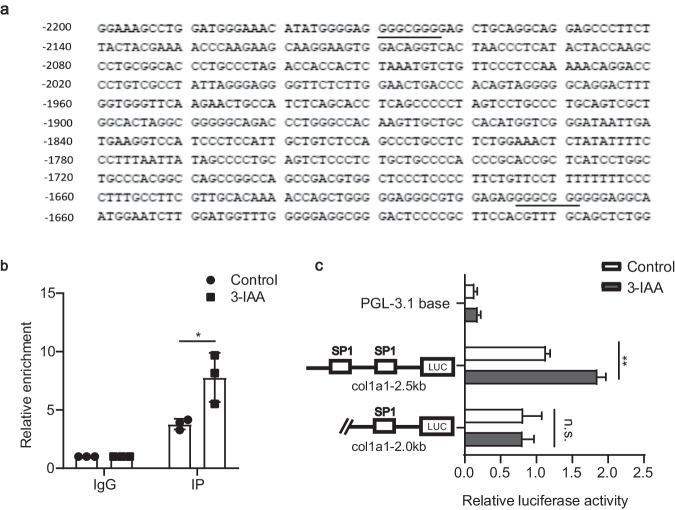
SP1 facilitates the activation of *COL1A1* after 3-IAA treatment. **a** The region –2300/–1600 of the human *COL1A1* gene was analyzed in Ensembl genome browser. The putative SP1 DNA binding sites are underlined. **b** HFSF cells were treated with PBS or 3-IAA (250 μM) for 24 h, prior to quantifying the percent enrichment of SP1 at the *COL1A1* gene using CUT&Tag-qPCR. **c** HFSF cells were transfected with luciferase constructs containing full length or truncated promoter elements of human *COL1A1* with wild-type putative SP1-binding sites or mutant-binding sites. After transfection, cells were treated with 3-IAA (250 μM) or PBS for 24 h. The data are presented as the mean ± SEM and evaluated using unpaired *t*-test (**b**, **c**). *n* = 3 (**b**, **c**). *P < 0.05, **P < 0.01. n.s. not significant.

We then investigated the mechanism of how 3-IAA regulates COL1A1 expression via SP1 in HFSFs. 2500-bp nucleotide sequence upstream of the *COL1A1* coding sequence was extracted and analyzed for the SP1 binding site using the JASPAR database (http://jaspar.genereg.net). We found two SP1 classical binding sites (GGGCGG) at −2169 bp to −2164 bp and −1614 bp to −1609 bp with the highest scores (Fig. 7a), which is in accordance with a previous study^22^. A CUT&Tag assay in HFSFs treated with 3-IAA or PBS vehicle was then performed. CUT&Tag-qPCR analysis showed a significant increase in the binding of SP1 to the *COL1A1* promoter region (Fig. 7b). Next, we constructed a firefly luciferase reporter driven by the promoter of the human *COL1A1* gene and found that *COL1A1* promoter activity was significantly up-regulated by 3-IAA. To further confirm the specificity of the binding site, we cut off the predicted binding site at −2000 bp. 3-IAA promoted the wild-type but not the truncated *COL1A1* promoter (Fig. 7c). Collectively, these results imply that 3-IAA treatment promotes *COL1A1* transcription by increasing the enrichment of SP1 at the promoter region.

## Discussion

**Fig. 8:**
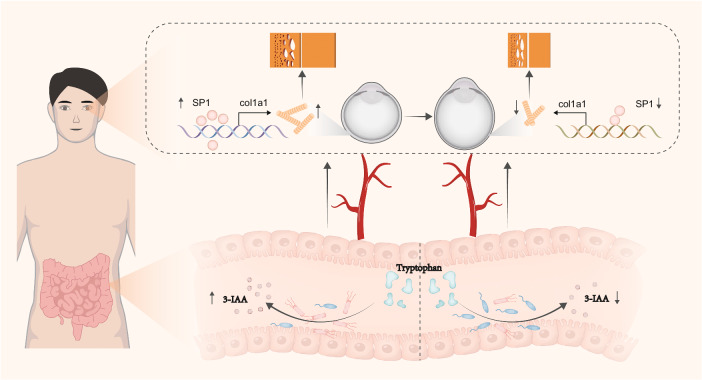
Gut microbiota regulates the progression of HM via the 3-IAA-SP1-COL1A1 pathway. In individuals with HM, dysbiosis in the gut microbiota leads to a decreased level of plasma 3-IAA. This reduction in 3-IAA impairs the binding of SP1 to the promoter region of *COL1A1* in the sclera, inhibiting its expression, leading to scleral thinning, and ultimately accelerating the progression of myopia.

HM is a leading cause of vision impairment and blindness worldwide. Despite numerous studies exploring its etiopathogenesis, the underlying mechanisms remain incompletely understood. Most studies have focused solely on ocular factors, neglecting its potential associations with systemic status. In our study, we identified significant alterations in the gut microbiota of HM individuals. FMT further demonstrated that the gut microbiota derived from HC effectively suppressed the progression of HM in mice. Notably, 3-IAA was the most significantly altered gut microbiota-associated metabolite in HM with oral supplementation in mice showing significantly elevated expression of COL1A1 in sclera and attenuated HM progression. Mechanistic explorations revealed that 3-IAA increased the expression of COL1A1 in an SP-1-dependent manner (Fig. 8).

HM development is associated with excessive axial elongation, which is accompanied by scleral ECM remodeling. This remodeling process usually involves a collagen decrease in ECM components, primarily COL1A1, which weakens the structural integrity of the sclera as shown in our results that COL1A1 decreased significantly after the HM induction^4^. Although numerous studies have investigated the mechanisms regulating COL1A1 expression, many have been limited to genetic factors, making intervention challenging. In recent years, researchers have discovered metabolic changes in both ocular tissues and circulation in HM individuals^14,16,23^, indicating a potential link between HM and systemic factors. Yet, how systemic conditions interfere with local collagen synthesis in HM development still remains unknown.

Mounting evidence indicates that gut microbiota can regulate collagen synthesis in fibrotic diseases such as those affecting the liver^24^, lung^25^, and kidney^9^ and restoring healthy microbial community homeostasis has been shown to improve prognosis^26,27^, indicating a possible gut-eye regulatory axis in collagen expression during HM development. In our study, by 16S rRNA gene sequencing, we did find significant differences in microbial community composition between HM and HC groups. Specifically, *Akkermansia*, *Parvimonas*, and *Gemella* were decreased, while *Butyricicoccus* and *Lautropia* were increased in the HM group. Thereinto, *Akkermansia* was the most decreased species identified in HM subjects and showed a significant inverse association with HM severity in terms of AL. *Akkermansia*, a next-generation beneficial microbe, has been found to ameliorate various disorders^14,28^, such as diabetes^29^, systemic lupus erythematosus^30^, and uveitis^31^ through the modulation of inflammation, which is also a known contributor to HM.

How gut microbiota changes in HM subjects may be possibly explained by HM-related lifestyles. Aberrant light exposure and disrupted circadian rhythms, being risk factors for myopia, are also known factors that may alter gut microbiota composition^32–34^. Interestingly, dim light could influence gut microbiota composition via intrinsically photosensitive retinal ganglion cells^35,36^, which is a novel subset of retinal ganglion cells that plays important role in the development of myopia^37^. Circadian rhythms were proved to regulate eye growth and were important factors that influenced the progression of myopia^38,39^. Aberrant light and circadian rhythms are common factors that affect both myopia and gut microbiota. One hypothesis is that these aberrant lifestyles not only directly contribute to HM but also reshape the gut microbiota, which in turn affects HM progression. To corroborate the causal relationship between HM and gut microbiota, we performed FMT in LIM mice and found that FMT from healthy controls significantly ameliorated the progression of HM. These results support that the development of HM may be intervened by regulating gut microbiota.

Bioactive metabolites are the main mediators that the gut microbiota generates, which can impact host physiology^40–42^. In our experiments, we identified seven significantly changed metabolites in HM patients, 3-IAA and indole-3-propionic acid are gut microbiota-associated tryptophan metabolites^42^. Among these, 3-IAA exhibited the most significant difference and showed correlations with both altered microbiota and clinical indices of myopia severity. These results suggest that it might be 3-IAA that mediates host-microbial crosstalk in HM patients. What’s more, oral administration of 3-IAA significantly retarded the progression of HM, and FMT_HC_ significantly increased plasma 3-IAA levels and scleral COL1A1 expression in mice, further confirming the close relationship between 3-IAA and gut microbiota in the development of HM. In our study, we excluded subjects with myopia-related complications at the time of sample collection to ensure a focused analysis of the primary effects of 3-IAA on myopia progression itself. Further studies targeting the effect of 3-IAA on these complications may be a promising research direction, potentially opening new therapeutic avenues to address a broader spectrum of myopia-related risks.

3-IAA is synthesized from indole-3-acetamide, which is converted from tryptophan by tryptophan 2-monooxygenase produced by bacteria. Previous studies have identified bacteria such as *Clostridium*, *Bacteroides*, and *Bifidobacterium* as the main producers of 3-IAA^43^. However, these bacteria did not change significantly in HM patients. Recent studies have reported that *Akkermansia* is also involved in tryptophan metabolism, which happens to be the species showing the most obvious reduction in HM individuals. Gu et al. found that both live *Akkermansia* and the functional outer protein of *Akkermansia* could significantly offset the reduction in 3-IAA levels in mice with colitis^19^. Yin et al. demonstrated that tryptophan treatment significantly promoted the growth of *Akkermansia*, which in turn metabolized tryptophan to indole, 3-IAA, indole-3-carboxaldehyde, and indole-3-lactic acid^20^. These findings highlight the crucial role of *Akkermansia* in tryptophan metabolism. Consistently, our experiments suggested that the gut microbiota of HM individuals, characterized by decreased *Akkermansia*, could influence plasma 3-IAA levels, indicating the potential role of *Akkermansia* in the progression of HM.

3-IAA is known as an endogenous AHR ligand that has been implicated in the regulation of fibrosis-related proteins, such as collagen, which is consistent with our finding of COL1A1 up-regulation by 3-IAA supplementation^21,43^. However, though the AHR signaling pathway could be activated by 3-IAA in HFSFs, we did not observe an abolishing effect of COL1A1 expression by AHR silencing. Mechanistic explorations identified that SP1, a transcriptional activator of COL1A1^22^ was significantly elevated after 3-IAA treatment in vitro. Luciferase assay further proved that 3-IAA facilitated the enrichment of the transcription factor SP1 in the distal promoter region of *COL1A1*, leading to the up-regulation of *COL1A1* transcription.

Currently, research on HM and gut microbiota is limited. Previously, in our tentative exploration, we found, for the first time, that the microbial composition and plasma metabolic profile differed considerably between HM mice and non-myopic mice and that the gut barrier integrity of HM mice was disrupted^44^. However, this study had certain limitations as it was solely based on findings from animal models, which, to a certain extent, compromised the clinical relevance of the results^44,45^. Besides, an observational study alone is insufficient to conclude whether HM leads to microbiome dysbiosis and metabolite alternations, or if it is the reverse. Hence, building upon these initial findings, we conducted a more comprehensive investigation. By starting with sample examinations from HM patients and implementing FMT and 3-IAA treatment, we intervened in the progression of HM in animal models. This approach supported the hypothesis that the altered gut microbiota composition leads to reduced plasma levels of 3-IAA, thereby contributing to HM progression. While our study identified a protective role for 3-IAA in lowering the progression of HM by assisting in maintaining the scleral expression of COL1A1, it is important to acknowledge that there might be multiple pathways beyond those discovered in our current research. Given its crucial role in regulating gut barrier function, 3-IAA could potentially serve as a mediator between gut function and HM^46^. However, whether it is a protective factor or a risk factor for gut barrier function remains a topic of debate in the current literature^47,48^. Thus, further investigation in this area is warranted.

In conclusion, all these findings allowed us to propose a novel conceptual advancement in understanding HM pathogenesis. HM is not solely an ocular morbidity but rather a result of the intricate interplay between the gut and the eye. The altered gut microbiota composition during HM progression causes unwanted declination of plasma 3-IAA which mechanistically helps maintain COL1A1 expression in sclera via a SP1-dependent transcriptional activation manner and suppress the progression of HM. More importantly, this identified microbiota-centric anti-HM effect of 3-IAA may open a new therapeutic avenue for HM intervention through daily supplementation.

## Materials and methods

### Human subjects

The study was reviewed and approved by the Eye & Ear, Nose, and Throat (EENT) Hospital of Fudan University, Shanghai, China (reference number: 2021145 for human research, 81470613 for animal research). All experiments adhered to the Declaration of Helsinki. Written informed consent was obtained from all participants. Animal experiments conform to the ARVO Statement.

The study recruited participants primarily from universities in Shanghai to ensure a homogeneous population with similar geographic locations and educational levels. HM was defined as AL ≥ 26.00 mm in both eyes, while HC were individuals with AL between 22.00 mm and 24.50 mm in both eyes. Exclusion criteria were as follows: (1) a history of surgery; (2) infectious- or immune-related eye diseases, such as uveitis, or any other ocular comorbidities (including cataracts, glaucoma, retinal detachment or tear, diabetic retinopathy, hypertensive retinopathy, age-related macular degeneration, retinitis pigmentosa, keratoconus, etc.; (3) any systemic disease reported during medical history taking; (4) a record of antibiotics, glucocorticoid, probiotics/prebiotics use within the past 3 months; (5) drug abuse or alcoholism.

A total of 97 subjects in the discovery cohort and 73 in an independent cohort were recruited after a rigorous diagnostic and exclusion process. The discovery cohort included 52 high myopes with 104 eyes and 45 HC with 90 eyes. The independent cohort included 26 high myopes with 52 eyes, 23 HC with 46 eyes, and 24 myopes with 24.50 ≤ AL <26.00 mm in both eyes. The validation set includes 26 high myopes and 23 HC from the independent cohort.

Personal information, including sex, age, educational level, weight, and height was recorded and BMI was calculated. AL measurement was performed by an experienced technician using the IOLMaster biometer (IOLMaster 700, Carl Zeiss, Germany).

### Acquisition, preservation, and usage of human samples

To evaluate the biochemical index of all participants, peripheral venous blood samples were collected in the fasting state. Tests included blood routine examination, including HbA1C, fasting blood glucose, triglyceride, and cholesterol, as well as hepatic and renal function analyses by automatic biochemical analyzers according to standardized national quality control protocols.

To analyze the plasma metabolome, 5 mL of peripheral venous blood samples were centrifuged (2000 rpm, 10 min) at 4 °C immediately after collection and then aliquoted into 1.5 mL microtubes. All samples were stored at –80 v until further analysis. A total of plasma samples from 39 HC and 49 HM subjects were used for targeted metabolomics analysis.

Participants were also instructed to fast overnight before feces collection. Fresh feces samples were collected using dedicated containers and divided into several 5 mL cryovials and were immediately stored at –80 °C until further 16S rRNA gene sequencing. For FMT, the fecal suspension was prepared as previously described. Briefly, feces were weighed and diluted with PBS to a final concentration of 150 mg/mL. The suspension was then filtered using a 40 μM filter and frozen at −80 °C with 20% glycerol until use.

### Mice

Male C57BL/6J mice were procured from Slack Laboratory Animal Co., Ltd. (China) and housed in specific pathogen-free conditions with food and water ad libitum at 21 °C under a 12-h/12-h light/dark cycle (with lights on at 7:00 AM and off at 7:00 PM) with 55%–60% humidity level.

### Lens-induced highly myopic mouse model

The procedure for inducing the highly myopic mouse model through lens manipulation has been previously described in detail^49^. Briefly, 4-week-old mice were used for induction of HM. A –30 D lens was affixed to the right eye, and the left eye served as the control. Daily inspections were conducted to ensure proper attachment of the lens. The refractive error of mice was measured on day 1 and day 21 using an infrared photorefractor (Steinbeis Transfer Center, Germany). AL was assessed using OCT (Towardpi, China) on Day 21. Each measurement was performed three times, and the mean value was used for subsequent analysis.

### FMT experiments

Mice were pretreated with ABX for one week to deplete their gut flora and were then used as recipients of FMT. ABX was prepared by combining broad-spectrum antibiotics, including ampicillin (0.25 mg/mL, A102048, Aladdin, China), metronidazole (0.25 mg/mL, M109874, Aladdin, China), neomycin (0.25 mg/mL, N432047, Aladdin, China) and vancomycin (0.125 mg/mL, V301569, Aladdin, China). The antibiotics were dissolved in autoclaved water and supplemented with sucralose (10 mg/mL, S432920, Aladdin, China). To confirm the successful establishment of the animal model, the bacterial counts in mouse feces were determined by 16S rRNA gene sequence. After a 2-day interim period following ABX treatment, the mice were administered 200 μL of thawed and mixed fecal suspension from either HC or HM human participants via oral gavage once a day for one week and once every two days for the following two weeks. Throughout the experiment, the weight, diopter, and AL of the mice were recorded.

### 3-IAA gavage

For 3-IAA supplementation, mice were administrated with 3-IAA solution (30 mg/kg/day) or water. Intragastric gavage was started 7 days before inducing HM using a 20 G flexible needle every day. The treatment continued until the mice were sacrificed.

### Acquisition, preservation, and usage of mouse samples

Mice were anesthetized with an intraperitoneal injection of pentobarbital sodium (50 mg/kg). The eyeballs were removed and blood was collected followed by centrifugation at 2000 rpm for 10 min at 4 °C. Scleras were carefully harvested from eye balls. The blood samples and tissues were frozen at −80 °C for further analysis.

### 16S rRNA gene sequencing and processing

Bacterial genomic DNA was extracted from fecal samples using the OMEGA Soil DNA Kit (M5635-02, Omega, USA). The quantity and quality of extracted DNA were assessed using a NanoDrop NC2000 spectrophotometer (ThermoFisher Scientific, USA) and agarose gel electrophoresis, respectively.

The bacterial 16S rRNA V3–V4 gene region was amplified using paired primers (338F/806R). The PCR amplicons were purified using Vazyme VAHTSTM DNA Clean Beads (N411-01, Vazyme, China) and quantified using the Quant-iT PicoGreen dsDNA Assay Kit (P7589, ThermoFisher Scientific, USA). The amplicons were then pooled in equal amounts and sequenced using the Illlumina NovaSeq platform (2 × 250, paired ends) with the NovaSeq 6000 SP Reagent Kit (20028312, Illumine, USA).

Briefly, raw data were demultiplexed using the demux plugin, followed by primers trimming with the cutadapt plugin. The sequences were denoised, quality-filtered, merged, and chimera was removed using the DADA2 plugin to obtain the amplicon sequence variants (ASVs) feature table. Non-singleton ASVs were aligned with MAFFT and used to construct a phylogeny using FastTree2. The sequences of all samples were rarefied to 23,999 sequences per sample for downstream diversity analysis.

Sequence data analyses were primarily performed using QIIME2 and R packages (v3.2.0). Differences in alpha diversity including Shannon’s index and the Chao1 index between groups were evaluated using the Kruskal–Wallis test. Beta diversity was assessed by PCoA. The significant differences between groups were tested using the Adonis test. LEfSe was employed to detect differentially abundant taxa across groups using the default parameters. The discriminating taxa were identified using a size-effect threshold of 2 on the logarithmic LDA score.

### Western blotting analysis

Proteins were obtained with RIPA lysis buffer (P0013B, Beyotime, China) which contained a protease inhibitor cocktail (11836153001, Roche, Switzerland), and were then separated using denaturing sodium dodecyl sulfate-polyacrylamide gel electrophoresis. Proteins were transferred onto polyvinylidene difluoride membranes (IPVH00010, Millipore, USA) followed by blocking with 5% milk at room temperature for 1 h and incubated with primary and secondary antibodies. Finally, the bands were visualized by Pierce Western Blotting Substrate Plus (ThermoFisher Scientific, USA). Band intensities were quantified using the ImageJ software (version 1.52) and normalized to β-actin levels. Antibodies against COL1A1(A1352, A22090 ABclonal, China), SP1 (21962-1-AP, Proteintech, China), AHR (28727-1-AP, Proteintech, China), β-actin (81115-1-RR, Proteintech, China) were used as primary antibodies. Goat anti-rabbit IgG-HRP (SA00001-2, Proteintech, China) was used as the secondary antibody.

### Immunohistochemistry

For immunohistochemistry staining, sections were incubated with 5 μg/mL proteinase K for 10 min to retrieve the antigens and were permeabilized in 0.3% Triton X-100 for 15 min followed by incubation in 2.5% methanolic hydrogen peroxide to block endogenous peroxidase activity. After washing, the sections were blocked with serum and incubated with the primary antibody overnight at 4 °C, and subsequently with biotinylated secondary antibody for 30 min at room temperature. The slides were then incubated with streptavidin-horseradish peroxidase. A DAB substrate was used to reveal the peroxidase activity. Slides were finally scanned by a digital scanner.

### Immunofluorescence

For immunofluorescence, cells were fixed for 10 min with 4% paraformaldehyde and permeabilized in 0.3% Triton X-100 for 15 min followed by blocking with immunofluorescence blocking buffer (P0102, Beyotime, China) for 1 h at room temperature. Then cells were probed with primary antibodies overnight at 4 °C, and with Alexa Fluor-488 labeled secondary antibody (A0423, Beyotime, China) for 1 h at room temperature. After being stained with DAPI, slides were visualized using confocal microscope (Leica Microsystems, Germany).

### Targeted plasma profiling

All standards of targeted metabolites were obtained from Sigma-Aldrich (St. Louis, MO, USA), Steraloids Inc. (Newport, RI, USA), and TRC Chemicals (Toronto, ON, Canada). 20 μL of plasma was vortexed with 120 μL of ice-cold methanol for 5 min. After centrifugation, 30 μL of supernatant was mixed with 20 μL of freshly prepared derivative reagents and incubated at 30 °C for 60 min. After derivatization, 330 μL of ice-cold 50% methanol solution was added to the sample and cooled at −20 °C for 20 min, followed by 4000 × *g* centrifugation at 4 °C for 30 min. Next, 135 μL supernatant was transferred to a new well and mixed with 10 μL internal standards.

The absolute quantification of plasma samples was performed by ultra-performance liquid chromatography coupled to tandem mass spectrometry (UPLC–MS/MS) system (ACQUITY UPLC-Xevo TQ-S, Waters Corp, Milford, MA, USA) and the analytical conditions were as follows: analytical column, ACQUITY UPLC BEH C18 (1.7 µM, 2.1 × 100 mm); elution solvents, water with 0.1% formic acid (A) and acetonitrile/IPA (70/30, v/v, B); gradient program, 0–1 min (5% B), 1 to 11 min (5%–78% B), 11–13.5 min (78%–95% B), 13.5–14 min (95%–100% B), 14–16 min (100% B), 16–16.1 min (100%–5% B), 16.1 to 18 min (5% B); injection volume, 5 μL; flow rate, 0.4 mL/min; column temperature, 40 °C; sample manager temp, 40 °C. The MS conditions were as follows: Capillary (Kv), 1.5 (ESI+) and 2.0 (ESI−); source temperature, 150 °C; desolvation temperature, 550 °C; desolvation gas flow, 1000 L/h. The raw data generated by UPLC–MS/MS were processed using the QuanMET software (v2.0, Metabo-Profile, Shanghai, China) to perform peak integration, calibration, and quantification for each metabolite. Internal standards, test mixtures, and pooled biological samples used in the metabolomics platform were used as quality control samples. In total, 193 metabolites were detected and used for further analysis.

### 3-IAA measurement

Mice were treated with 3-IAA or water for one week by intragastric administration. Ocular enucleation was performed 3 h after the final gavage. Scleral tissues from five mice were pooled to formed a single sample with each experimental group comprising three such samples. Briefly, each scleral sample was homogenized by adding 10 times its weight of methanol/water for testing (1:1, v/v). The homogenates were mixed with methanol and centrifuged (12,000 rpm at 4 °C for 5 min). After centrifugation, all the supernatant was transferred to a new tube and dried using a vacuum concentrator. The remnants were then redissolved using 50 μL methanol/water (1:1, v/v). Finally, 5 μL of the solution was used for the subsequent LC–MS/MS analysis.

For plasma samples, 100 μL plasma was mixed with an equal volume of methanol and centrifuged at 10,000 rpm at 4 °C for 10 min. The 100 μL supernatant was collected into a sample bottle and 5 μL was used for the subsequent LC–MS/MS analysis.

The LC–MS/MS analysis was performed using an API 6500 Qtrap Mass Spectrograph (Sciex, USA) coupled to hydrophilic interaction chromatography via electrospray ionization at the Institutes of Biomedical Sciences, Fudan University. LC separation was performed on an Ultimate 3000 UHPLC (ThermoFisher Scientific, USA) with ultimate AQ-C18 column (2.1 × 250 mm, 5 μm particle size) using a gradient of solvent A (0.1% formic acid solution) and solvent B (0.1% formic acid-acetonitrile solution). The gradient started with 0% solvent B for 1 min and then linearly increased to 90% over 2 min and maintained for 4 min, reduced to 0% over 0.1 min, and maintained for 7.9 min. The flow rate was 0.4 mL/min. The mass spectrometer was conducted in positive ionization modes. Electron spray ionization conditions were set as follows: curtain gas, 40 psi; ion source gas 1, 40 psi; ion source gas 2, 40 psi; collision gas, medium; ionspray voltage, 5000 V and temperature of 500 °C. For the quantification of 3-IAA, a standard curve for the concentration ranges from 1.6 fmol/μL to 25 pmol/μL was constructed. Data was processed using skyline (v4.2).

### Cell culture and treatments

HFSFs were obtained from Cell Resource Center, IBMS, CAMS/PUMC (Beijing, China) and cultured in DMEM medium supplemented with 10% fetal bovine serum (FBS). Cells were incubated at 37 °C in a normal humidified atmosphere with 5% CO_2_.

3-IAA (I2886, Sigma)stock solution was prepared by completely dissolving it in NaOH (1 N) and followed by adding 25% (v/v) HCl to adjust the pH to 7.4. To explore the effects of 3-IAA on HFSFs, cells were preincubated with different concentrations of 3-IAA for 24 h/48 h following a 6-h period of FBS starvation. After the treatment, cells were collected for further experiments.

### RNA isolation and real-time PCR

Total RNA was extracted using the EZ-press RNA Purification Kit (B0004D, EZBioscience, China) according to the manufacturer’s instructions. RNA was quantified by a Nanodrop spectrophotometer (ThermoFisher Scientific, USA) and was then inversed into cDNA using the HiFiScript gDNA Removal RT MasterMix kit (CW2020, CWBIO, China). Real-time PCR was performed with MagicSYBR Mixture (CW3008, CWBIO, China) in a Real-Time PCR System (BioRad, USA). The primer sequences for the genes are shown in Supplementary Table S3.

### CUT&Tag assay

Briefly, HFSF cells were treated with either PBS (control) or 3-IAA (500 μm) for 24 h in six-well plates. The NovoNGS ChiTag Transposon Kit (N259-YH01-01A, Novoprotein) was used in this assay. The cells were harvested and treated according to the manufacturer’s instructions. Anti-SP1 antibody (21962-1-AP, Proteintech, China) was used to pull down the DNA-protein complexes, while the rabbit IgG was used as a negative control. The purified DNA were subjected to PCR. The information on the human *COL1A1* promoter (−2169 bp to −2164 bp) primers is as follows: Forward-5′CCTTGGAGGTTTCAACTCTT3′, Reverse-5′GTTTCCCATCCAGGCTTT3′.

### Luciferase assay

For promoter activity assay, HFSFs were seeded in 96-well plates and co-transfected with col1a1-Luc or renilla plasmid using Lipofectamine 3000 (L3000015, ThermoFisher Scientific, USA) for 24 h. Cells were then treated with 3-IAA (500 μM) or its vehicle for 24 h. The cells were then harvested for measuring firefly and renilla luciferases using the Dual-Luciferase Reporter Assay System (11402ES60, Yeasen, China).

### MR analysis

To investigate potential causal relationships between gut microbiota and myopia risk, a two-sample MR analysis was performed. Public large-scale GWAS data for gut microbiota downloaded from the GWAS Catalog (GCST90027446 - GCST90027857, N = 7738)^17^ were used as exposure data. GWAS summary statistics for myopia publicly available from the FinnGen study (finn-b-H7_MYOPIA, N_case_ = 1640, N_control_ = 210,931) were used as outcome data. The following criteria were applied to select genetic instruments for all exposure data: (1) GWAS *P*-value < 1 × 10^−5^; (2) linkage disequilibrium (LD) r^2^ < 0.001 and linkage disequilibrium distance > 10,000 kb; (3) The F statistic å 10. The Steiger direction test was used to test the causal direction of each instrumental variable. All the SNPs used for analysis were checked in LDlink (https://ldlink.nci.nih.gov/?tab=ldtrai) to minimize the confounders. In this study, We conducted MR analysis by four methods (IVW, MR-Egger regression, Weighted median, and Weighted mode) using the“TwoSampleMR” R package. The IVW method was the main method used to estimate associations between gut microbiota traits and myopia risk. Cochrane’s Q method and MR-Egger intercept were used to test the heterogeneity and potential horizontal pleiotropy of the MR analysis respectively.

### Statistics and reproducibility

Statistical analyses were performed using GraphPad Prism v8, and R v3.3.2. For normally distributed data, a paired two-tailed *t*-test was employed to test the statistical significance between two eyes of the same animal, whereas unpaired *t*-test, one-way ANOVA or two-way ANOVA followed by Bonferroni’s post-hoc tests were applied when comparing eyes from different groups. The Wilcoxon rank-sum test was used if the variables were inconsistent with the normal distribution. Spearman’s correlation *P*-values were adjusted for multiple comparisons using the Benjamini–Hochberg false discovery rate. The chi-square test and Fisher’s exact test were used to compare categorical variables. A *P*-value of < 0.05 was considered significant.

### Supplementary information

SUPPLEMENTAL MATERIAL
