# Supplementary material for: Gut microbiota-derived indole-3-acetic acid suppresses high myopia progression by promoting type I collagen synthesis
Source: Cell Discov. 2024 Aug 27;10:89. doi: 10.1038/s41421-024-00709-5 (PMC11347609; doi:10.1038/s41421-024-00709-5)
Supplement: Supplementary file 1 — SUPPLEMENTAL MATERIAL [file 41421_2024_709_MOESM1_ESM.docx]

| Characteristics | n = 24 |
| --- | --- |
| age (years) | 25.7 ± 2.94 |
| Males/Females n (%) | 11 (45.8)/13 (54.2) |
| Educational level  （a/b/c/d/e/f/g） | 0/0/0/0/12/10/2 |
| BMI (kg/m^2^) | 21.43 (19.81, 23.27) |
| AL (mm）-OD | 25.23 ± 0.39 |
| AL (mm）-OS | 25.19 ± 0.49 |
| ACD (mm)-OD  ACD (mm)-OS  LT (mm)-OD  LT (mm)-OS  WTW (mm)-OD  WTW (mm)-OS  Diopter(D)-OD  Diopter(D)-OS  IOP (mmHg)-OD  IOP (mmHg)-OS | 3.71 ± 0.24  3.71 ± 0.25  3.65 ± 0.27  3.59 ± 0.27  12.00 ± 0.33  12.03 ± 0.32  -4.01 ± 1.38  -3.86 ± 1.39  15.25 ± 3.06  15.33 ± 2.67 |
| FPG (mmol/L) | 4.71 (4.33, 5.03) |
| TG (mmol/L) | 0.87 ± 0.29 |
| TC (mmol/L) | 4.48 ± 0.94 |
| AST(U/L) | 18.42(16.00,19.50) |
| ALT(U/L) | 18.71(9.00,21.00) |
| HbA1C (%) | 5.45±0.20 |
| Cr(μmol/L) | 67.75±13.77 |
| UA(μmol/L) | 339.80±116.10 |

**Supplementary Table S1**

Data are presented as mean ± standard deviation (SD), median with interquartile range (IQR), or n (%). P-values are based on the two-sided t test for variables expressed as mean ± SD, Wilcoxon rank-sum test for variables expressed as median (IQR) and chi-square test for variables expressed as percentages. HC healthy control, HM high myopia; educational level (a/b/c/d/e/f/g): primary education/lower secondary education /upper secondary or intermediate vocational education / higher vocational education/ undergraduate education/ postgraduate education/ doctoral education; BMI: Body Mass Index; AL: Axial Length; ACD: Anterior Chamber Depth; LT: Lens Thickness; WTW: White to White; IOP: Intraocular Pressure; FPG: Fasting Plasm Glucose; TG: Triglyceride; TC: Total Cholesterol; AST: Aspartate Transaminase; ALT: Alanine Transaminase Cr: creatinine; UA: Uric Acid**Supplementary Table S2**

| **Exposure** | **Outcome** | **Method** | **Nsnp** | **OR** | **OR (95% CI)** | **beta** | **se** | **P.pleiotropy** | **P.heterogeneity** | **P.MR** |
| --- | --- | --- | --- | --- | --- | --- | --- | --- | --- | --- |
| **g_Akkermansia** | **Myopia** | **IVW** | **12** | **0.797** | **0.637-** **0.997** | **-0.227** | **0.114** | **0.939** | **0.977** | **0.047** |
| **g_Akkermansia** | **Myopia** | **MR Egger** | **12** | **0.766** | **0.271-2.166** | **-0.267** | **0.531** |  | **0.942** | **0.626** |
| **g_Akkermansia** | **Myopia** | **Weighted median** | **12** | **0.794** | **0.587-1.073** | **-0.231** | **0.154** |  |  | **0.133** |
| **g_Akkermansia** | **Myopia** | **Weighted mode** | **12** | **0.828** | **0.488-1.404** | **-0.189** | **0.270** |  |  | **0.498** |
| **g_Sutterellaceae** | **Myopia** | **IVW** | **5** | **1.310** | **1.010-1.698** | **0.270** | **0.132** | **0.804** | **0.977** | **0.042** |
| **g_Sutterellaceae** | **Myopia** | **MR Egger** | **5** | **1.483** | **0.581-3.783** | **0.394** | **0.478** |  | **0.942** | **0.470** |
| **g_Sutterellaceae** | **Myopia** | **Weighted median** | **5** | **1.300** | **0.941-1.797** | **0.263** | **0.165** |  |  | **0.111** |
| **g_Sutterellaceae** | **Myopia** | **Weighted mode** | **5** | **1.341** | **0.897-2.004** | **0.293** | **0.205** |  |  | **0.225** |
| **g_Haemophilus** | **Myopia** | **IVW** | **4** | **0.703** | **0.540-0.916** | **-0.352** | **0.135** | **0.893** | **0.778** | **0.009** |
| **g_Haemophilus** | **Myopia** | **MR Egger** | **4** | **0.814** | **0.121-5.492** | **-0.206** | **0.974** |  | **0.585** | **0.852** |
| **g_Haemophilus** | **Myopia** | **Weighted median** | **4** | **0.703** | **0.506-0.976** | **-0.352** | **0.167** |  |  | **0.035** |
| **g_Haemophilus** | **Myopia** | **Weighted mode** | **4** | **0.691** | **0.454-1.052** | **-0.370** | **0.214** |  |  | **0.183** |
| **s_Oxalobacter_formigenes** | **Myopia** | **IVW** | **7** | **0.773** | **0.615-0.974** | **-0.257** | **0.117** | **0.315** | **0.461** | **0.029** |
| **s_Oxalobacter_formigenes** | **Myopia** | **MR Egger** | **7** | **0.381** | **0.108-1.348** | **-0.966** | **0.645** |  | **0.491** | **0.195** |
| **s_Oxalobacter_formigenes** | **Myopia** | **Weighted median** | **7** | **0.721** | **0.530-0.982** | **-0.327** | **0.157** |  |  | **0.038** |
| **s_Oxalobacter_formigenes** | **Myopia** | **Weighted mode** | **7** | **0.702** | **0.470-1.047** | **-0.354** | **0.204** |  |  | **0.134** |
| **s_Haemophilus_parainfluenzae** | **Myopia** | **IVW** | **5** | **0.712** | **0.559-0.907** | **-0.339** | **0.123** | **0.880** | **0.899** | **0.006** |
| **s_Haemophilus_parainfluenzae** | **Myopia** | **MR Egger** | **5** | **0.807** | **0.177-3.676** | **-0.214** | **0.774** |  | **0.790** | **0.780** |
| **s_Haemophilus_parainfluenzae** | **Myopia** | **Weighted median** | **5** | **0.727** | **0.533-0.993** | **-0.319** | **0.159** |  |  | **0.045** |
| **s_Haemophilus_parainfluenzae** | **Myopia** | **Weighted mode** | **5** | **0.726** | **0.495-1.065** | **-0.320** | **0.195** |  |  | **0.177** |
| **s_Coprococcus_sp_ART55_1** | **Myopia** | **IVW** | **3** | **1.256** | **1.015-1.553** | **0.228** | **0.108** | **0.817** | **0.576** | **0.036** |
| **s_Coprococcus_sp_ART55_1** | **Myopia** | **MR Egger** | **3** | **1.369** | **0.743-2.520** | **0.314** | **0.311** |  | **0.314** | **0.497** |
| **s_Coprococcus_sp_ART55_1** | **Myopia** | **Weighted median** | **3** | **1.218** | **0.942-1.576** | **0.197** | **0.131** |  |  | **0.133** |
| **s_Coprococcus_sp_ART55_1** | **Myopia** | **Weighted mode** | **3** | **1.161** | **0.856-1.576** | **0.149** | **0.156** |  |  | **0.439** |
| **f_Prevotellaceae** | **Myopia** | **IVW** | **8** | **1.460** | **1.108-1.922** | **0.378** | **0.141** | **0.745** | **0.854** | **0.007** |
| **f_Prevotellaceae** | **Myopia** | **MR Egger** | **8** | **1.720** | **0.642-4.606** | **0.542** | **0.503** |  | **0.783** | **0.322** |
| **f_Prevotellaceae** | **Myopia** | **Weighted median** | **8** | **1.457** | **1.003-2.117** | **0.377** | **0.191** |  |  | **0.048** |
| **f_Prevotellaceae** | **Myopia** | **Weighted mode** | **8** | **1.472** | **0.870-2.490** | **0.386** | **0.268** |  |  | **0.193** |
| **f_Oxalobacteraceae** | **Myopia** | **IVW** | **7** | **0.774** | **0.615-0.974** | **-0.256** | **0.117** | **0.324** | **0.458** | **0.029** |
| **f_Oxalobacteraceae** | **Myopia** | **MR Egger** | **7** | **0.387** | **0.109-1.372** | **-0.950** | **0.646** |  | **0.480** | **0.201** |
| **f_Oxalobacteraceae** | **Myopia** | **Weighted median** | **7** | **0.722** | **0.530-0.982** | **-0.326** | **0.157** |  |  | **0.038** |
| **f_Oxalobacteraceae** | **Myopia** | **Weighted mode** | **7** | **0.701** | **0.466-1.055** | **-0.355** | **0.208** |  |  | **0.140** |
| **f_Pasteurellaceae** | **Myopia** | **IVW** | **3** | **0.716** | **0.522-0.982** | **-0.334** | **0.161** | **0.926** | **0.956** | **0.038** |
| **f_Pasteurellaceae** | **Myopia** | **MR Egger** | **3** | **0.630** | **0.071-5.570** | **-0.462** | **1.112** |  | **0.781** | **0.749** |
| **f_Pasteurellaceae** | **Myopia** | **Weighted median** | **3** | **0.724** | **0.490-1.068** | **-0.324** | **0.199** |  |  | **0.104** |
| **f_Pasteurellaceae** | **Myopia** | **Weighted mode** | **3** | **0.729** | **0.476-1.117** | **-0.315** | **0.217** |  |  | **0.284** |
| **f_Akkermansia** | **Myopia** | **IVW** | **12** | **0.797** | **0.637-** **0.998** | **-0.226** | **0.114** | **0.942** | **0.870** | **0.048** |
| **f_Akkermansia** | **Myopia** | **MR Egger** | **12** | **0.767** | **0.272-2.165** | **-0.265** | **0.529** |  | **0.812** | **0.627** |
| **f_Akkermansia** | **Myopia** | **Weighted median** | **12** | **0.794** | **0.590-1.069** | **-0.231** | **0.152** |  |  | **0.128** |
| **f_Akkermansia** | **Myopia** | **Weighted mode** | **12** | **0.827** | **0.484-1.416** | **-0.189** | **0.274** |  |  | **0.504** |
| **o_Pasteurellales** | **Myopia** | **IVW** | **3** | **0.716** | **0.522-0.982** | **-0.334** | **0.161** | **0.926** | **0.956** | **0.038** |
| **o_Pasteurellales** | **Myopia** | **MR Egger** | **3** | **0.630** | **0.071-5.565** | **-0.462** | **1.112** |  | **0.781** | **0.749** |
| **o_Pasteurellales** | **Myopia** | **Weighted median** | **3** | **0.724** | **0.497-1.054** | **-0.324** | **0.192** |  |  | **0.092** |
| **o_Pasteurellales** | **Myopia** | **Weighted mode** | **3** | **0.730** | **0.456-1.168** | **-0.315** | **0.240** |  |  | **0.319** |
| **o_Verrucomicrobiales** | **Myopia** | **IVW** | **12** | **0.797** | **0.637-** **0.998** | **-0.226** | **0.114** | **0.942** | **0.870** | **0.048** |
| **o_Verrucomicrobiales** | **Myopia** | **MR Egger** | **12** | **0.767** | **0.272-2.165** | **-0.265** | **0.529** |  | **0.812** | **0.627** |
| **o_Verrucomicrobiales** | **Myopia** | **Weighted median** | **12** | **0.794** | **0.592-1.064** | **-0.231** | **0.150** |  |  | **0.123** |
| **o_Verrucomicrobiales** | **Myopia** | **Weighted mode** | **12** | **0.827** | **0.496-1.379** | **-0.189** | **0.261** |  |  | **0.483** |
| **p_Firmicutes** | **Myopia** | **IVW** | **6** | **1.511** | **1.048-2.179** | **0.413** | **0.187** | **0.285** | **0.760** | **0.027** |
| **p_Firmicutes** | **Myopia** | **MR Egger** | **6** | **0.659** | **0.168-2.586** | **-0.417** | **0.698** |  | **0.896** | **0.582** |
| **p_Firmicutes** | **Myopia** | **Weighted median** | **6** | **1.301** | **0.816-2.073** | **0.263** | **0.238** |  |  | **0.268** |
| **p_Firmicutes** | **Myopia** | **Weighted mode** | **6** | **1.225** | **0.624-2.406** | **0.203** | **0.344** |  |  | **0.581** |
| **p_Verrucomicrobia** | **Myopia** | **IVW** | **12** | **0.798** | **0.637-** **0.998** | **-0.226** | **0.114** | **0.942** | **0.870** | **0.048** |
| **p_Verrucomicrobia** | **Myopia** | **MR Egger** | **12** | **0.767** | **0.272-2.165** | **-0.265** | **0.529** |  | **0.812** | **0.628** |
| **p_Verrucomicrobia** | **Myopia** | **Weighted median** | **12** | **0.794** | **0.581-1.085** | **-0.231** | **0.160** |  |  | **0.148** |
| **p_Verrucomicrobia** | **Myopia** | **Weighted mode** | **12** | **0.827** | **0.488-1.402** | **-0.189** | **0.269** |  |  | **0.496** |

**Supplementary Table S3**

| Gene | Forward primer | Reverse primer |
| --- | --- | --- |
| c-fos | GCAGCGAACGAGCAGTGACC | GAGAACATCATCGTGGCGGTTAGG |
| c-jun | AAGAACTCGGACCTCCTCACCTC | GCCCGTTGCTGGACTGGATTATC |
| smad4 | CAGCCAGGACAGCAGCAGAATG | TGGTGGTGAGGCAAATTAGGTGTG |
| smad3 | AGGACACAGGAAGAGACGGAAGG | CACACCAGGCACATACTTCAGACTC |
| smad2 | AATTCTTCCTTCAGCACCGCCTTAG | ACCAACACCAAACAGCACCTCAG |
| sp3 | GTCAGCAGATGGTCAGCAGGTTC | GAAGGTGTTCCAGAGGCAAGTAAGG |
| sp1 | CTGCCACCATGAGCGACCAAG | GCTACTGCCTGTGCTGCTACTTC |
| col1a2 | CCGTGGCAGTGATGGAAGTGTG | GCAGGACCAGCGTTACCAACAG |
| tgf-b1 | TATTGAGCACCTTGGGCACTGTTG | CCTTAACCTCTCTGGGCTTGTTTCC |
| cyp1a1 | TTCGCTACCTACCCAACCCTTCC | GTAGTGCTCCTTGACCATCTTCTGC |
| col1a1 | TGGCAAAGAAGGCGGCAAAGG | AGGAGCACCAGCAGGACCATC |
| actin | GGCCAACCGCGAGAAGATGAC | GGATAGCACAGCCTGGATAGCAAC |
| tgf-b2 | TGCTGCCTACGTCCACTTTACATTG | CCTGCTGTGCTGAGTGTCTGAAC |
| bmp2 | TCCCGACAGAACTCAGTGCTATCTC | GACACCCACAACCCTCCACAAC |


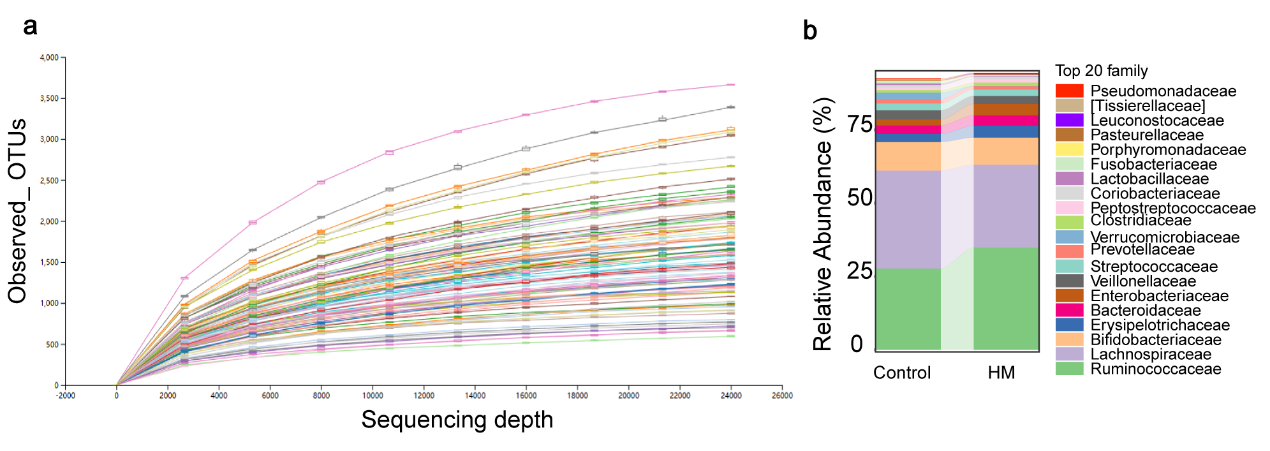


**Supplemental Fig. S1**

**a** Rarefaction curves of 97 samples from HC and HM subjects. **b** Family level of microbial composition (top 20).


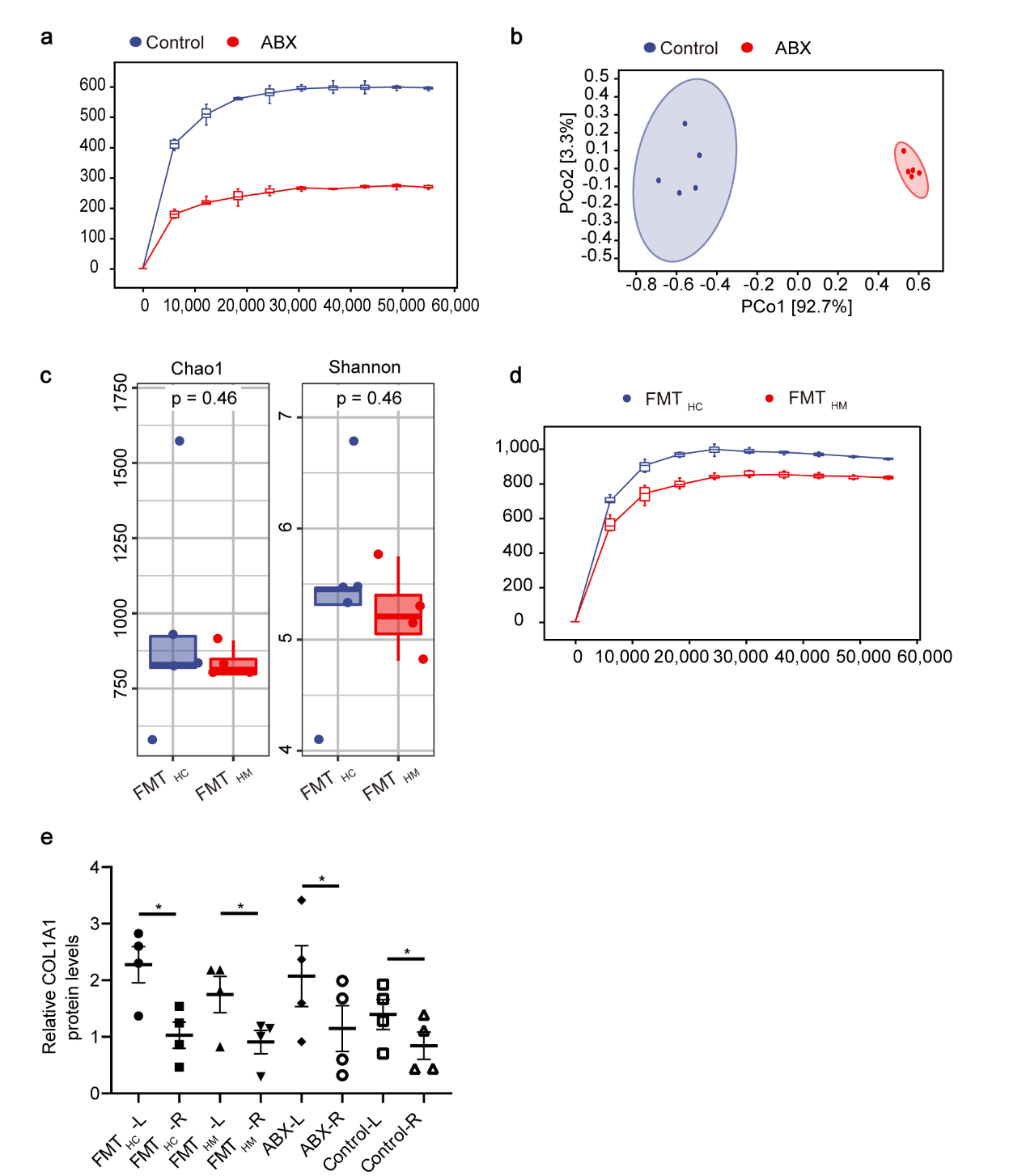


**Supplemental Fig. S2**

**a** Rarefaction curves of control or ABX-treated mice based on Chao1 index. **b** PCoA plot based on the weighted UniFrac distance. **c** The diversity and abundance of the gut microbiota in FMT_HC_ and FMT_HM_-treated mice were assessed by Chao1 (*P* = 0.46) and Shannon index (*P* = 0.46). **d** Rarefaction curves of FMT_HC_ or FMT_HM_-treated mice based on Chao1 index. **e** Densitometric quantification of blots in Fig. 2i using ImageJ. n = 5 (**a, b**). n = 4 (FMT_HC_ group in **c** and **d**) and n = 4 (FMT_HM_ group in **c** and **d**), n = 4 (**e**, each sample pooled 3 sclerae). The data are presented as median, quartiles, and min/max values (**a**, **c**, **d**) or the mean ± SEM evaluated using unpaired two-tailed t-test (**d**). *P*-values are evaluated by Wilcoxon rank-sum test (**c**) or paired two-tailed t-test (**e**). * *P* < 0.05.


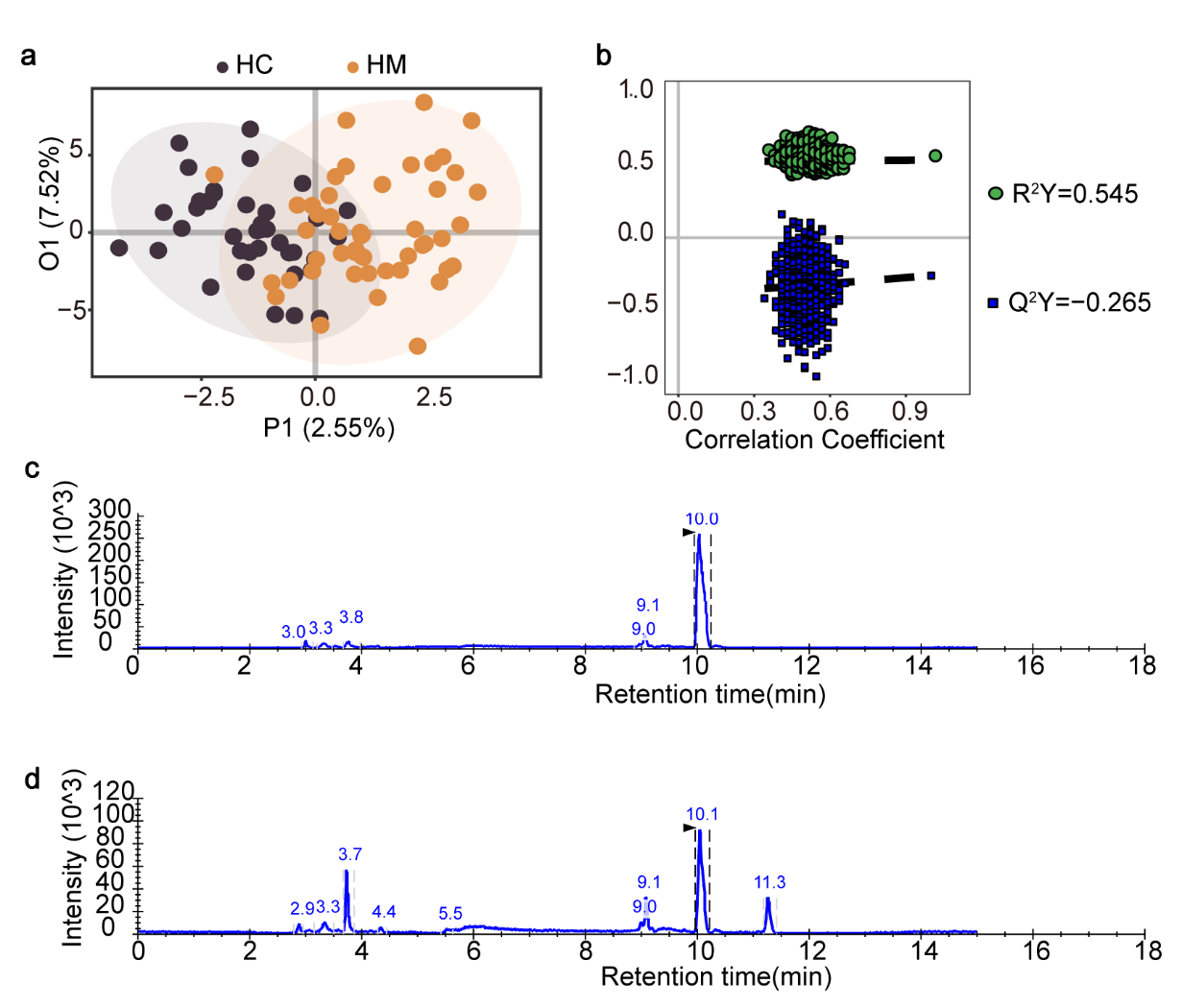


**Supplemental Fig. S3**

**a** OPLS-DA showing the differential clustering patterns of metabolites in plasma between HM and HC subjects. **b** Permutation test of the OPLS-DA model. Representative chromatogram of 3-IAA in mouse plasma in FMT_HC_ group (**c**) or FMT_HM_ group (**d**).


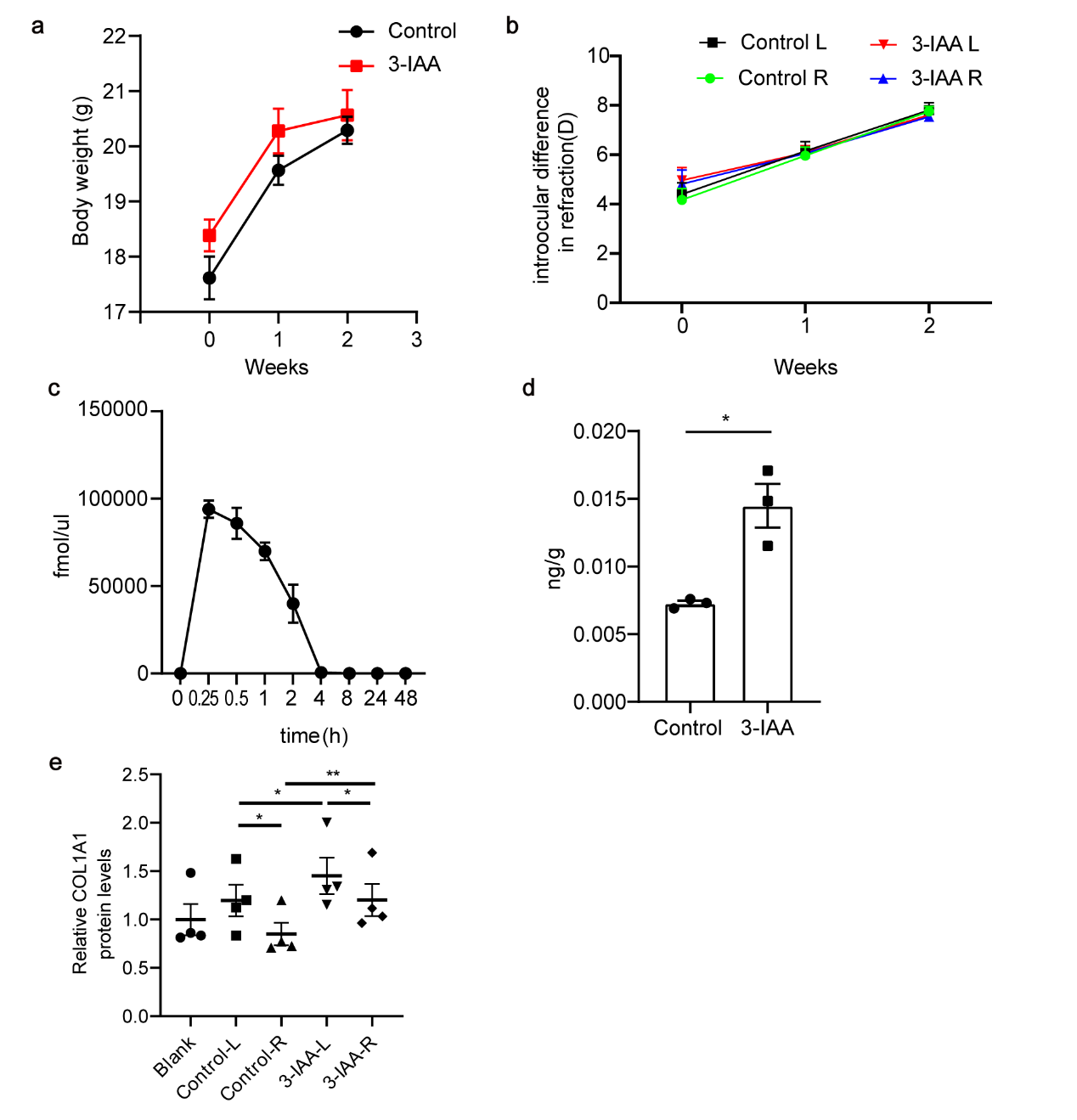


**Supplemental Fig. S4**

**a** Changes in body weight in response to 3-IAA daily gavage in C57BL/6 mice. **b** Effect of daily 3-IAA gavage on refraction during mice ocular development. n = 8 (**a**, **b**). **c** 3-IAA plasma concentration of wild type mice supplemented with 30 mg/kg of 3-IAA were measured using LC-MS/MS at 15min (n=4), 30min (n=4), 2h (n=3), 4h (n=4), 8h (n=4), 24h (n=4) and 48h (n=4) after 3-IAA application. (**d**) 3-IAA concentration of the sclera in wild type mice supplemented with30 mg/kg 3-IAA at 3 hours after 3-IAA application. n = 3 (each sample pooled 10 sclerae). (**e**) Densitometric quantification of blots in Fig. 4g using ImageJ. The data are presented as the mean ± SEM and evaluated using unpaired two-tailed t-test (**d**), paired two-tailed t-test (**e**, comparison of L and R eyes in each treatment), and two-way ANOVA followed by Bonferroni’s post hoc tests (**a**, **b** and the comparison of different groups in e). * *P* < 0.05, ***P* < 0.01.


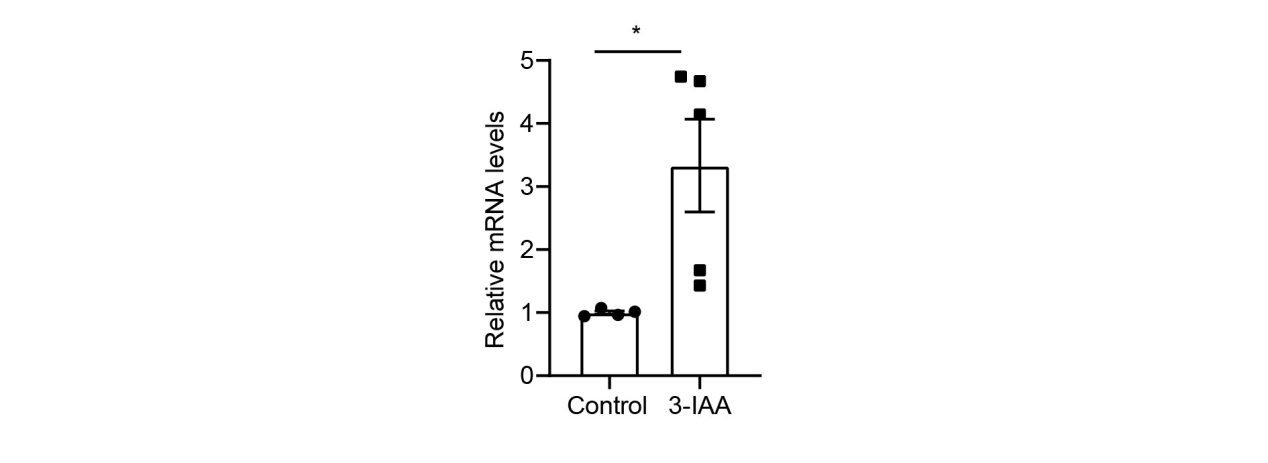


**Supplemental Fig. S5**

mRNA levels of *CYP1A1* in PBS or 3-IAA-treated HFSF cells. n = 5. The data are presented as the mean ± SEM and evaluated using unpaired two-tailed t-test. * *P* < 0.05.


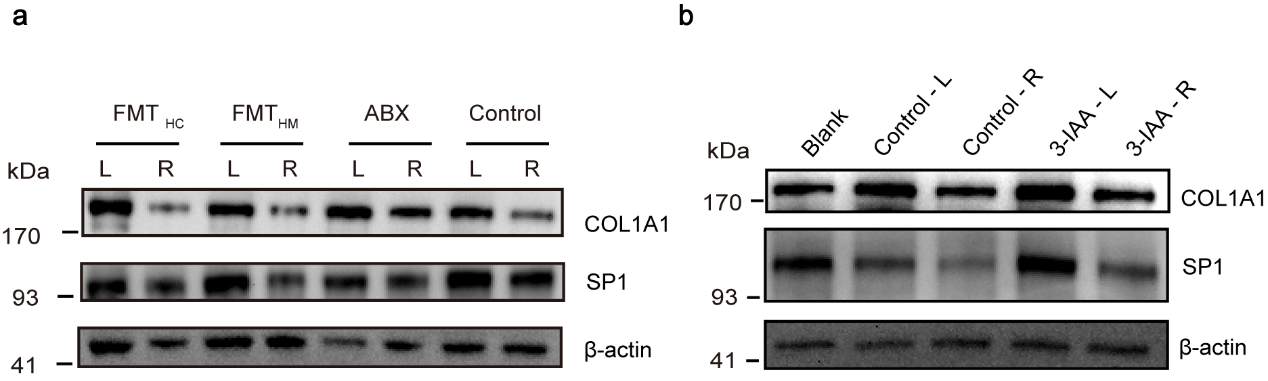


**Supplemental Fig. S6**

The protein levels of COL1A1 and SP1 in the sclera of FMT mice (**a**) or 3-IAA/ vehicle treatment mice (**b**) were determined by western blotting. β-actin was used as the control.
